# Guiding *Clostridioides difficile* Infection Prevention Efforts in a Hospital Setting With AI

**DOI:** 10.1001/jamanetworkopen.2025.15213

**Published:** 2025-06-12

**Authors:** Shengpu Tang, Stephanie Shepard, Rebekah Clark, Erkin Ötleş, Chidimma Udegbunam, Josh Tran, Melinda Seiler, Justin Ortwine, Akbar K. Waljee, Jerod Nagel, Sarah L. Krein, Jacob E. Kurlander, Paul J. Grant, Jihoon Baang, Anastasia Wasylyshyn, Krishna Rao, Jenna Wiens

**Affiliations:** 1 Department of Computer Science, Emory College of Arts and Sciences, Emory University, Atlanta, Georgia; 2 Department of Electrical Engineering and Computer Science, Division of Computer Science and Engineering, University of Michigan College of Engineering, Ann Arbor; 3 Department of Emergency Medicine, University of Wisconsin School of Medicine and Public Health, Madison; 4 Center for Healthcare Engineering & Patient Safety (CHEPS), University of Michigan College of Engineering, Ann Arbor; 5 Health Information Technology & Services (HITS), Michigan Medicine, Ann Arbor; 6 AI & Digital Health Innovation, Michigan Medicine, Ann Arbor; 7 Department of Learning Health Sciences, University of Michigan Medical School, Ann Arbor; 8 Department of Internal Medicine, Division of Gastroenterology & Hepatology, University of Michigan Medical School, Ann Arbor; 9 Department of Pharmacy, Michigan Medicine, Ann Arbor; 10 Center for Clinical Management Research, Veterans Affairs Ann Arbor Healthcare System, Ann Arbor, Michigan; 11 Department of Internal Medicine, Division of General Medicine, University of Michigan Medical School, Ann Arbor; 12 Department of Internal Medicine, Division of Hospital Medicine, University of Michigan Medical School, Ann Arbor; 13 Office of Clinical Informatics, Michigan Medicine, Ann Arbor; 14 Department of Internal Medicine, Division of Infectious Diseases, University of Michigan Medical School, Ann Arbor

## Abstract

**Importance:**

Increasingly, artificial intelligence (AI) is being used to develop models that can identify patients at high risk for adverse outcomes. However, the clinical impact of these models remains largely unrealized.

**Objective:**

To evaluate the association of an AI-guided infection prevention bundle with *Clostridioides difficile* infection (CDI) incidence in a hospital setting.

**Design, Setting, and Participants:**

This prospective, single-center quality improvement study evaluated adult inpatient hospitalizations before (September 1, 2021, to August 31, 2022) and after (January 1, 2023, to December 31, 2023) AI implementation. Data analysis was performed from January to August 2024.

**Intervention:**

A previously validated institution-specific AI model for CDI risk prediction was integrated into clinical workflows at the study site. The model was used to guide infection prevention practices for reducing pathogen exposure through enhanced hand hygiene and reducing host susceptibility through antimicrobial stewardship.

**Main Outcomes and Measures:**

The primary outcome was CDI incidence rate. Secondary outcomes included antimicrobial use and qualitative assessments of bundle implementation.

**Results:**

Pre-AI and post-AI samples included 39 046 (21 645 [55.4%] female; median [IQR] age, 58 [36-70] years) and 40 515 (22 575 [55.7%] female; median [IQR] age, 58 [37-70] years) hospitalizations, respectively. After adjusting for differences in clinical characteristics, there was no significant reduction in CDI incidence (pre-AI period: 5.76 per 10 000 patient-days vs post-AI period: 5.65 per 10 000 patient-days; absolute difference, −0.11; 95% CI, −1.43 to 1.18; *P* = .85). Relative reductions greater than 10% in normalized antimicrobial days were seen for piperacillin-tazobactam (−9.64; 95% CI, −12.93 to −6.28; *P* < .001) and clindamycin (−1.04; 95% CI, −1.60 to −0.47; *P* = .03), especially for high-risk patients alerted by AI (relative reduction for piperacillin-tazobactam, 16.8%; 95% CI, 8.0%-24.6%). On the basis of qualitative assessments via semistructured interviews and field observations, the study found that health care staff’s experiences with AI-guided workflows varied. In particular, the enhanced hand hygiene protocols were met with poor adherence, whereas pharmacists consistently engaged with the alerts.

**Conclusions and Relevance:**

In this quality improvement study, the implementation of an AI-guided infection prevention bundle was not associated with a significant reduction in the already low CDI incidence rate at the study site, but it was associated with reduced CDI-associated antimicrobial use. The results highlight the potential of AI in supporting antimicrobial stewardship. Barriers to implementation, including infrastructure, staff knowledge, and workflow integration, need to be addressed in future applications.

## Key Points

**Question:**

Can artificial intelligence (AI) tools effectively guide infection prevention efforts in hospitals?

**Findings:**

In this quality improvement study with 39 046 participants in the pre-AI period and 40 515 participants in the post-AI period, implementation of an AI-guided infection prevention bundle was not associated with a significant reduction in *Clostridioides difficile* infection (CDI) incidence (the primary outcome) but was associated with substantial reductions in CDI-associated antimicrobial use.

**Meaning:**

AI tools may support CDI prevention and antimicrobial stewardship in hospitals, but success depends on thoughtful and effective integration into clinical workflows.

## Teaser

This quality improvement study examines a 28-month quality improvement program that used an institution-specific AI model to guide *Clostridioides difficile* infection prevention efforts at a large academic medical center.

## Introduction

The Centers for Disease Control and Prevention (CDC) has identified *Clostridioides difficile* as an urgent antibiotic resistance threat in the US.^1,2^ *Clostridioides difficile* infection (CDI), one of the most common health care–associated infections, is associated with substantial morbidity, mortality, and increased hospital costs.^3,4^ Susceptible patients develop CDI when exposed to the pathogen, either from health care personnel who have touched contaminated surfaces^5,6^ or from the community before admission.^7^ Given that *Clostridioides difficile* forms spores and is resistant to alcohol-based hand sanitizers,^8^ effective CDI prevention often includes soap-and-water handwashing. Moreover, receipt of certain antimicrobials, including clindamycin, ampicillin, and carbapenems, is associated with increased susceptibility.^9,10,11^ Thus, antimicrobial stewardship is also key to preventing CDI.^12,13,14,15^

However, applying these practices at scale is challenging because the number needed to treat to prevent 1 CDI case can be unfavorably high given resource constraints at most hospitals.^16,17^ Targeting prevention efforts on patients most likely to acquire CDI could prove more effective. To this end, numerous artificial intelligence (AI) models have been developed to predict CDI risk in hospitalized patients with the goal of identifying high-risk patients who may potentially benefit from targeted infection prevention resources.^18,19,20,21,22,23^ Although these models have shown promise in prospective validation^24,25^ and in identifying patients prior to colonization,^26^ their association with patient outcomes in clinical care remains unknown.^24^

In this article, we report the results of a 28-month quality improvement program in which we used an institution-specific AI model to guide CDI prevention efforts at a large academic medical center. Using data from electronic health records (EHRs), we compared patient outcomes before and after AI implementation, including CDI rates and antimicrobial stewardship performance measures.

## Methods

The University of Michigan Medical School institutional review board deemed this study to be an unregulated quality improvement project and approved the supplemental implementation evaluation of various intervention components with a waiver of informed consent from patients and authorization for accessing protected health information from EHR databases. We followed the Standards for Quality Improvement Reporting Excellence (SQUIRE) reporting guideline for quality improvement studies.

### Study Design and Participants

In this quality improvement study with a pre-post quasi-experimental design, we assessed whether an AI-guided infection prevention bundle was associated with a reduction in CDI incidence at Michigan Medicine (MM), the academic medical center affiliated with the University of Michigan. Our study sample included adult inpatient hospitalizations (admission age ≥18 years) from September 1, 2021, through December 31, 2023 (Figure). We excluded psychiatric patients and hospitalizations lasting 24 hours or less. Patient self-reported race and ethnicity were extracted from the EHR to assess sample diversity and representativeness. Race categories included Asian, Black, White, and other (American Indian or Alaska Native, Native Hawaiian and Other Pacific Islander, other, unknown, patient refused, or missing but not recorded as Asian, Black, or White). Ethnicity categories included Hispanic or Latino, non-Hispanic or non-Latino, and other (patient refused, unknown, or missing). The pre-AI period was from September 1, 2021, to August 31, 2022, and the post-AI period was from January 1, 2023, to December 31, 2023. The 4-month implementation rollout period from September 1, 2022, to December 31, 2022, was excluded from analysis.

**Figure.**
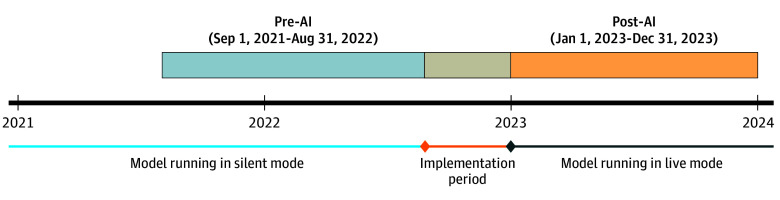
Study Design and Date Ranges for the Samples Before and After Artificial Intelligence (AI) Implementation The 4-month implementation rollout period from September 1, 2022, to December 31, 2022, was excluded from analysis.

### Model Specification

The CDI Risk Score is calculated by an L2-regularized logistic regression model with time-varying parameters, built and described previously.^20,24,25,27^ The model leverages routinely collected EHR data (including patient demographics, laboratory tests, vital signs, comorbidities, and medications) to estimate the daily risk of a patient acquiring CDI in the remainder of their hospitalization. On a patient cohort admitted in 2021, the model achieved an area under the receiver operating characteristic curve of 0.822 (95% CI, 0.804-0.838) (eMethods and eTable 1 in Supplement 1). Prior to morning rounds (typically 7-10 am), the model calculated risk scores for all patients hospitalized for more than 24 hours who had not tested positive for *Clostridioides difficile*, sent those scores to the EHR, and triggered the infection prevention bundle for high-risk patients.

### Infection Prevention Bundle Design

During several months from late 2021 until the implementation period, the study team engaged in conversations with various stakeholders, including hospitalists, nursing teams, pharmacists, antimicrobial stewardship, patient engagement, and the clinical informatics office, to discuss the design of the infection prevention bundle. Key decisions pertained to frequency and timing of alerts in the context of current workflows (to prevent alert fatigue), recipients of alerts, and modes of delivery of various bundle components. Based on historical CDI incidence, we selected 10 hospital units to focus our implementation efforts where they were most likely to have an impact; they included medical intensive care units, surgical intensive care units, medical wards, surgical wards, hematopoietic stem cell transplant wards, and step-down units. We performed a simulation analysis and selected an alert threshold based on the 91st percentile of historical risk scores, leading to a sensitivity of 31%, a precision of 5 times the baseline incidence rate, and an expected alert frequency of 5 alerts per week per hospital unit on average (eMethods and eFigures 1 and 2 in Supplement 1). With this configuration, we anticipated the study to have 95% power to detect a reduction in CDI incidence assuming the bundle was 30% or more effective at preventing CDI (eFigure 1 in Supplement 1).

The infection prevention bundle was applied to high-risk patients and aimed to reduce both pathogen exposure and host susceptibility (eFigure 3 in Supplement 1). Given each patient’s daily score trajectory, as soon as the score exceeded the alert threshold, the patient was considered high risk and deemed eligible for our bundle in the remainder of hospitalization. If a patient tested positive for *Clostridioides difficile* during hospitalization (by polymerase chain reaction [PCR]), standard CDI contact precautions superseded our bundle. The bundle included components across 3 distinct modes of care delivery: (1) best practice advisory alerts (BPAs), (2) medication reviews by inpatient pharmacists, and (3) dedicated health record reviews by physicians on the study team. We provide a brief overview here with additional figures and justifications in eFigures 4 to 8 in Supplement 1.

(1) Two BPAs were displayed when the patient’s primary inpatient practitioner opened the record. BPA 1 (eFigure 4 in Supplement 1) asked the practitioner to place a nursing order for enhanced hand hygiene. After the order, the nursing team would display a sign on the door of the patient’s room (eFigure 5 in Supplement 1) that instructed all persons entering the room to wash their hands with soap and water upon entry instead of using alcohol-based hand sanitizers. These patients would receive an informational flyer (eFigure 6 in Supplement 1) that explained our initiative. BPA 2 (eFigure 7 in Supplement 1) suggested a list of strategies to reduce CDI risk, including discontinuing unnecessary antibiotics or acid suppressants, recommending the patient eat yogurt (if appropriate), and consulting the β-lactam allergy evaluation service for patients who reported β-lactam allergies.^28^ (2) The CDI risk score was converted to a binary high-risk flag and displayed in an existing sorting column of pharmacist medication review dashboard (eFigure 8 in Supplement 1). Pharmacists used this flag to sort and filter patients, documenting communications with the patient’s care team and/or medication changes as Epic iVents (Epic Systems). Based on feedback from the pharmacy team, this component made use of the same aforementioned score threshold but was implemented hospital-wide given the centralized nature of pharmacists’ workflows. (3) Dedicated medical record reviews were conducted on business days for the patients with highest risk by physicians from the study team (A.W., J.H.B., K.R., and P.J.G.), who communicated specific recommendations (eg, de-escalating antibiotics) directly to the patients’ care teams. Of these, BPA 1 aimed to directly reduce pathogen exposure by preventing anyone entering the patient’s room from bringing in the pathogen. All remaining bundle components aimed to reduce host susceptibility, primarily through improved antimicrobial stewardship and minimizing unnecessary acid suppression therapy.

### Patient-Oriented Outcomes

The primary outcome was incidence of laboratory-identified CDI. During the study period, MM used a testing algorithm that first conducts a PCR test and reflexes to a toxin test by enzyme immunoassay (EIA) only if the PCR test result is positive. Laboratory-identified CDI included test results that were positive by both PCR and EIA. We assessed other measures of laboratory testing, including testing rates, rates of PCR-positive CDI (regardless of toxin result), and rates of hospital-onset CDI. We also measured the association with patients’ medication use, specifically antibiotics and acid suppressants. We developed EHR-based outcome definitions using data from the MM Research Data Warehouse, with appropriate denominators per reporting guidelines from CDC National Healthcare Safety Network.^29,30,31^ See eMethods in Supplement 1 for details.

### Implementation Assessment

To understand perceptions and use of the infection prevention bundle, we conducted semistructured interviews with health care personnel who interacted with each bundle component.^35,36^ Candidate interviewees were identified and recruited from the list of BPA recipients and inpatient pharmacists. The interview questions focused on general impressions as well as role-specific workflow impacts. To assess adherence to the enhanced hand hygiene sign, we conducted 2 rounds of field observations. In June to July 2023, we recorded whether health care staff entering patient rooms with the signs in fact washed their hands before entering. In December 2023, we recorded whether signs were posted in accordance with the BPA. Analysis of implementation-related data was primarily descriptive: we generated statistics of health care personnel’s interactions with bundle components and used a rapid-analysis process to compile and summarize the interview and observation data (eMethods in Supplement 1). Interview protocols and field observation recording templates are provided as eAppendixes 1 and 2 in Supplement 1.

### Statistical Analysis

We first quantified differences between pre- and post-AI samples in demographic and clinical characteristics. Patient-level data were queried from the EHR. We conducted *z* tests for comparing proportions of binary variables, χ^2^ tests for homogeneity for comparing categorical variables, and Mann-Whitney *U* tests for comparing continuous variables, with a Bonferroni correction to adjust for multiple hypotheses. For outcome comparisons, we controlled for differences in sample characteristics via a 1:1 nearest-neighbor matching. Specifically, we matched the pre-AI sample to the post-AI distribution to facilitate outcome comparisons that are representative of the expected future distribution, estimating the average treatment effect on the treated (eMethods in Supplement 1).^32^ Similar to previous work,^33^ we accounted for potential confounding by adjusting for a range of covariates, including age, biological sex, self-identified race and ethnicity, admission months and location, whether the hospitalization was related to COVID-19, and recent hospitalization history. We computed 95% CIs of outcomes and pre-post differences using 1000 bootstrapped samples. Statistical significance of outcome differences was determined using 2-sided resampling tests on the bootstraps.^34^ A 2-sided *P* < .05 was considered statistically significant. A Bonferroni correction was applied to *P* values for secondary outcomes.

## Results

### Sample Characteristics

Pre- and post-AI samples included 39 046 (21 645 [55.4%] female and 17 399 [44.6%] male; 1192 [3.1%] Asian, 5330 [13.7%] Black, 30 753 [78.8%] White, and 2034 [5.2%] other; median [IQR] age, 58 [36-70] years) and 40 515 (22 575 [55.7%] female and 17 939 [44.3%] male; 1262 [3.1%] Asian, 5219 [12.9%] Black, 32 231 [79.6%] White, and 2105 [5.2%] other; median [IQR] age, 58 [37-70] years) hospitalizations, respectively, and differed along several dimensions, including age, COVID-19–related hospitalizations, and recent hospitalization history (Table 1; eFigure 9 and eTable 2 in Supplement 1). Although the differences appear numerically small, many of them are statistically significant and have known associations with CDI risk. Thus, in subsequent sections, we report adjusted results to control for these potential sources of confounding (unadjusted results are provided as eTable 3 in Supplement 1).

**Table 1.** Characteristics of the Pre-AI and Post-AI Samples

Characteristic	No. (%) of participants	
	Pre-AI (n = 39 046)	Post-AI (n = 40 515)
**Demographic characteristics**		
Sex		
Female	21 645 (55.4)	22 575 (55.7)
Male	17 399 (44.6)	17 939 (44.3)
Unknown	2 (<0.01)	1 (<0.01)
Age at admission, median (IQR), y	58 (36-70)	58 (37-70)
Age group at admission, y		
18-25	2963 (7.6)	2853 (7.0)
26-45	10 958 (28.1)	11 032 (27.2)
46-65	11 808 (30.2)	12 222 (30.2)
66-85	11 933 (30.6)	13 019 (32.1)
>85	1384 (3.5)	1389 (3.4)
Race^a^^,^^b^		
Asian	1192 (3.1)	1262 (3.1)
Black	5330 (13.7)	5219 (12.9)
White	30 753 (78.8)	32 231 (79.6)
Other^c^	2034 (5.2)	2105 (5.2)
Ethnicity^a^		
Hispanic or Latino	1374 (3.5)	1503 (3.7)
Non-Hispanic or non-Latino	36 815 (94.3)	37 968 (93.7)
Other^d^	857 (2.2)	1044 (2.6)
**Clinical characteristics**		
Month of admission^e^		
Jan-Apr	13 063 (33.5)	13 117 (32.4)
May-Aug	12 896 (33.0)	14 166 (35.0)
Sep-Dec	13 087 (33.5)	13 232 (32.7)
Length of stay, median (IQR), d	3 (2-7)	3 (2-7)
COVID-19–associated hospitalization^f^	2517 (6.4)	975 (2.4)
Recent hospitalization^g^	10 204 (26.1)	13 652 (33.7)
Recent medication exposure^f^		
Antimicrobials	6176 (15.8)	7082 (17.5)
Acid suppressants	5483 (14.0)	5673 (14.0)
Recent comorbidity history^g^^,^^h^		
Acute myocardial infarction	1031 (2.6)	1238 (3.1)
Congestive heart failure	2440 (6.2)	2946 (7.3)
Peripheral vascular disease	1720 (4.4)	2182 (5.4)
Chronic obstructive pulmonary disease	2291 (5.9)	3222 (8.0)
Diabetes without complications	2302 (5.9)	2880 (7.1)
Diabetes with complications	1900 (4.9)	2017 (5.0)
Kidney disease	2711 (6.9)	3378 (8.3)
Cancer, any malignant neoplasm	2749 (7.0)	3485 (8.6)
Metastatic solid tumor	1116 (2.9)	1311 (3.2)
Inflammatory bowel disease	326 (0.8)	402 (1.0)
Admission location^i^		
Medical ICUs	1060 (2.7)	1072 (2.6)
Surgical ICUs	2973 (7.6)	3201 (7.9)
Step-down units	410 (1.1)	639 (1.6)
Medical wards	12 557 (32.2)	12 644 (31.2)
Medical-surgical wards	4002 (10.2)	4171 (10.3)
Surgical wards	7729 (19.8)	8375 (20.7)
Oncology wards	2056 (5.3)	2079 (5.1)
HSCT wards	1387 (3.6)	1162 (2.9)
Maternity wards	5760 (14.8)	5644 (13.9)
Overflow wards	396 (1.0)	820 (2.0)
Other wards^j^	716 (1.8)	708 (1.7)

Abbreviations: AI, artificial intelligence; HSCT, hematopoietic stem cell transplant; ICU, intensive care unit.

^a^ Race and ethnicity were reported separately to reflect the data entry practice during the study period.

^b^ Percentages for race add up to more than 100% because the electronic health record allows for selection of multiple races.

^c^ Other race includes patients recorded as American Indian or Alaska Native, Native Hawaiian and Other Pacific Islander, other, unknown, patient refused, or missing but not recorded as Asian, Black, or White.

^d^ Other ethnicity includes patient refused, unknown, and missing.

^e^ Months of admission were grouped into 4-month periods that were contiguous date ranges for both pre- and post-AI samples.

^f^ Defined as those who had either a positive laboratory test result for SARS-CoV-2 or a recorded *International Statistical Classification of Diseases and Related Health Problems, Tenth Revision (ICD-10) *diagnosis code for COVID-19. Alternative definitions considered in eTable 2 in Supplement 1.

^g^ Recent was defined as those recorded in the previous 90-day encounters.

^h^ See eTable 2 in Supplement 1 for an extended comparison of recent comorbidity history.

^i^ Admission location was defined as the first inpatient location of a hospitalization (excluding emergency departments or observational wards).

^j^ Other admission location includes stroke wards, burn wards, and rehabilitation wards.

### Outcomes

There were 127 CDI cases for 255 017 patient-days in the pre-AI period and 148 CDI cases for 261 892 patient-days in the post-AI period (eTable 4 in Supplement 1). The adjusted incidence CDI rate per 10 000 patient-days was 5.76 (95% CI, 4.87-6.69) in the pre-AI period and 5.65 (95% CI, 4.78-6.56) in the post-AI period (absolute difference, −0.11; 95% CI, −1.43 to 1.18; *P* = .85) (Table 2). There was no difference in other laboratory testing measures but significant reductions in the use of ampicillin-sulbactam (−2.82; 95% CI, −4.59 to −1.03; *P* = .03), piperacillin-tazobactam (−9.64; 95% CI, −12.93 to −6.28; *P* < .001), and clindamycin (−1.04; 95% CI, −1.60 to −0.47; *P* = .03), measured in antibiotic days per 1000 days present (Table 2). Reductions were more prominent in high-risk hospitalizations alerted by the AI tool (eg, piperacillin-tazobactam: relative reduction of 16.8% [95% CI, 8.0%-24.6%; *P* < .001] in high risk vs no significant change in low risk) (eTables 5 and 6 in Supplement 1). Utilization rates of other medications we considered did not change significantly.

**Table 2.** Primary and Secondary Outcomes Comparison Between Adjusted Pre-AI and Post-AI Samples

Outcome	Estimate (95% CI)		Change (95% CI)	*P *value^a^
	Pre-AI (adjusted)	Post-AI		
Primary outcome				
*Clostridioides difficile* infections, incidence rate per 10 000 patient-days^b^	5.76 (4.87 to 6.69)	5.65 (4.78 to 6.56)	−0.11 (−1.43 to 1.18)	.85
Secondary outcomes				
Laboratory testing of CDI, instances per 10 000 patient-days				
Test conducted	119.89 (116.17 to 123.73)	115.05 (111.36 to 118.92)	−4.84 (−10.16 to 0.81)	>.99
PCR-positive *Clostridioides difficile* test^c^	18.61 (17.04 to 20.24)	18.10 (16.49 to 19.75)	−0.51 (−2.89 to 1.75)	>.99
Hospital-onset CDI^d^	3.98 (3.24 to 4.78)	3.74 (2.99 to 4.54)	−0.24 (−1.36 to 0.83)	>.99
Antibiotic use, days of therapy per 1000 d present				
Ampicillin-sulbactam	20.45 (19.22 to 21.73)	17.63 (16.42 to 18.87)	−2.82 (−4.59 to −1.03)	.03
Piperacillin-tazobactam	59.21 (56.76 to 61.50)	49.58 (47.28 to 52.00)	−9.64 (−12.93 to −6.28)	<.001
Ceftriaxone	19.20 (17.61 to 20.84)	18.01 (16.65 to 19.41)	−1.19 (−3.28 to 1.03)	>.99
Cefepime, concurrent with metronidazole	9.00 (8.16 to 9.94)	10.98 (9.82 to 12.15)	1.98 (0.52 to 3.43)	.24
Carbapenems^e^	16.05 (14.48 to 17.73)	16.90 (15.27 to 18.56)	0.85 (−1.43 to 3.13)	>.99
Clindamycin	3.38 (2.97 to 3.85)	2.34 (1.98 to 2.73)	−1.04 (−1.60 to −0.47)	.03
Fluoroquinolones^f^	26.44 (24.57 to 28.43)	28.32 (26.44 to 30.17)	1.88 (−0.90 to 4.43)	>.99
Acid suppressant use, days of therapy per 1000 d present				
Proton pump inhibitors^g^	255.69 (250.21 to 261.71)	249.94 (244.20 to 255.43)	−5.74 (−14.36 to 2.28)	>.99
Histamine_2_-blockers^h^	114.49 (109.95 to 118.91)	110.49 (106.12 to 114.83)	−4.01 (−10.24 to 2.58)	>.99

Abbreviations: AI, artificial intelligence; CDI, *Clostridioides difficile* infection; PCR, polymerase chain reaction.

^a^ *P* values for secondary outcomes are for 2-sided tests and corrected with a Bonferroni adjustment.

^b^ Laboratory-identified CDI.

^c^ Includes all tests with positive results for PCR regardless of enzyme immunoassay result (could be positive or negative).

^d^ Subset of laboratory-identified CDI that are hospital onset.

^e^ Carbapenems include meropenem, imipenem, and ertapenem.

^f^ Fluoroquinolones include ciprofloxacin, moxifloxacin, and levofloxacin.

^g^ Proton pump inhibitors include omeprazole, lansoprazole, pantoprazole, and esomeprazole.

^h^ Histamine_2_-blockers include famotidine, cimetidine, and nizatidine.

### Implementation Assessment

Descriptive statistics for key infection prevention bundle components are discussed in the eResults and eFigure 10 in Supplement 1. Of the 17 primary inpatient practitioners interviewed, 6 indicated that they tended to ignore the BPAs and 10 could not recall the BPA content. Selected quotations from practitioners about different bundle components they interacted with are given in Table 3. Field observations revealed an overall low adherence rate for the enhanced hand hygiene sign (eResults in Supplement 1). However, of the 7 pharmacists interviewed, 6 indicated the new workflow was not disruptive, and all 7 contacted the patients’ care team based on the alerts (eTables 7 and 8 in Supplement 1).

**Table 3.** Select Interview Quotations From Primary Inpatient Practitioners for Key Infection Prevention Bundle Components^a^

Bundle component	Selected quotation
Best practice alerts	[Interviewer: Can you describe how you would generally proceed after receiving an alert?] “I just see the alert and, I mean, we have like pharmacists and things that go through the medications and make sure that whatever antibiotics somebody’s on are the right ones. So, I tend to, just, you know, see them, acknowledge it, know, to wash my hands when I go in the room, and then I kind of like dismiss them. I’ve never went ahead and like ordered anything from the order set to be honest.” (Interviewee 8, resident)
Handwashing with soap and water	[Interviewer: What would you say are the biggest challenges with handwashing on room entry?] “The biggest challenges...Well, everyone’s got a sink in their room, even if it’s in the bathroom. So, I don’t think that that’s too big of an issue. Probably the biggest challenge that I feel that most people would say would just be time.” (Interviewee 4, physician assistant) [Interviewer: What are the biggest challenges with handwashing on room entry?] “I think it’s just that the sink is not available right outside of the room.” (Interviewee 5, physician assistant)
Communication among pharmacist, study team physician, and care team	[Interviewer: What do you think about the calls from pharmacists or study team physicians?] “[...] sometimes that’s been a good flag of like, Hey, this is the time to probably narrow antibiotics or rethink things. So, I will change. But I mean, obviously sometimes I’m busy and I see this a bit of a nuisance, but usually it’s somewhat helpful. And I don’t always change antibiotics, but at least will like change my documentation if I’m not [clear] or something.” (Interviewee 10, resident)
Reduce or discontinue antibiotics or PPIs	[Interviewer: What did you think of the calls from pharmacists when you did get them?] “I think [the calls from pharmacy are] reasonable. I understand why, they give me the reason why they do it. I would say that sometimes it is kinda annoying, because, and I understand why they’re doing it, but I have my thought processes, too, of why they have to stay on PPI. For example. I don’t know if you know this, but lung transplant patient they have a lot of acid reflux, and we actually want to prevent it because it causes higher risk for rejection. So, some of our people, even if there’s high *C diff* risk, when you talk about rejection and potentially not having a lung work any longer, which is life threatening, you just gotta pick what’s more beneficial.” (Interviewee 5, physician assistant)
Recommend yogurt	[Interviewer: Have you ever encouraged a patient identified as high risk for *C diff* to eat yogurt?] “No, no, but I think I know probiotics was kind – it’s kind of a thing. So that kind of intuitively makes sense. But we never said yogurt.” (Interviewee 18, resident)
BLAES consultation	[Interviewer: Have you placed a β-lactam allergy consult after receiving an alert?] “No, not after receiving an alert, I think for a different reason.” (Interviewee 3, resident)

Abbreviations: BLAES, β-lactam allergy evaluation service; PPI, proton pump inhibitor.

^a^ The interview protocols and specific questions being asked can be found in eAppendix 1 in Supplement 1.

## Discussion

In this prospective quality improvement study, we used an AI tool to guide hospital infection prevention practices by focusing on patients at highest risk of acquiring CDI. Our infection prevention efforts addressed modifiable CDI risk factors related to both pathogen exposure and host susceptibility, such as encouraging the practice of hand hygiene with soap-and-water handwashing and prompting dialogues on meaningful changes in antimicrobial therapy. Although we did not observe a significant reduction in CDI incidence, we observed significant reductions in the use of CDI-associated antimicrobials.

In the past decade, AI has been increasingly used to develop institution-specific risk prediction models for clinical outcomes.^37,38,39^ Despite this enormous potential and increasing interest in the health care AI community, the clinical impact of most models remains largely unrealized.^40^ The lack of appropriate technological infrastructures has often been cited among the primary roadblocks.^41,42^ Although we also encountered infrastructure challenges, we found the implementation challenges related to clinical integration to be far greater. Despite significant efforts that went into designing the infection prevention bundle, we observed limited and potentially inadequate use of some bundle components by health care staff. In particular, our attempt to reduce exposure through enhanced hand hygiene practices was not successful.

The failure of the handwashing component can be attributed to 2 primary factors. First, despite our careful design choices to prevent alert fatigue (on average, a practitioner received only 1 or 2 of our BPAs per week), this was in a broader context of high alert volume—practitioners already encounter an estimated 6 to 8 alerts (beyond the ones in this study) per day that require a response. As a result, there were still signs of alert fatigue as reflected by the interviews, indicating that many practitioners simply ignored the BPAs; in an already saturated alert environment, even well-designed alerts may struggle to stand out. Second, following through the enhanced hand hygiene component required health care personnel to adopt new behaviors at multiple stages: practitioners needed to respond to a new BPA, nurses needed to put up the signage, and all clinical staff were asked to wash their hands with soap and water instead of standard alcohol-based hand sanitizer use. This multistage behavior change created a so-called leaky pipeline: BPAs were often ignored, and orders for enhanced hand hygiene were not created; even when orders were placed, the signs were not always posted or followed. As a result, imperfect adherence at each stage compounded and led to overall poor adherence.

In contrast, our efforts had the greatest association with antimicrobial stewardship, likely because the AI guidance was integrated into current workflows of pharmacists. Rather than introducing multistage new behaviors, the tool helped pharmacists prioritize higher-risk patients in their existing work. Among our interviewees, almost all pharmacists reported performing additional medication reviews as indicated by the high-risk flag, engaging in conversations with care teams about de-escalating antimicrobials and acid suppression when appropriate. This finding highlights the importance of good implementation science that integrates behavior changes seamlessly into existing workflows.^43,44,45^ Although this was feasible for the pharmacists because their work is centralized and our education and engagement efforts can reach everyone involved, achieving the same for enhanced hand hygiene was more difficult due to its decentralized nature and the need for continual education, especially in an academic hospital setting with regularly rotating practitioners.

Our study results should be considered in the context of the maximum achievable benefit assuming perfect implementation. The already low baseline rates of CDI at MM, with fewer than 200 cases annually, presented a challenge for any infection prevention effort to lead to substantial improvement. Furthermore, even though exposure and susceptibility are the primary drivers for infectious diseases, reducing them is not always possible. For example, some patients might already be colonized with *Clostridioides difficile* on admission, and others might require specific antimicrobials (eg, carbapenems) or acid suppression treatments that could not be altered.

### Limitations

Our study has limitations. First, it was conducted at a single tertiary health care center, meaning the performance characteristics and outcome measures are institution specific and dependent on clinical epidemiology and variations in the patient population. However, the challenges encountered and the lessons learned are generalizable to other institutions looking to integrate AI models into clinical practice. Moreover, our study was not a randomized clinical trial; the quasi-experimental analysis design was deliberate. Randomization at the patient or practitioner level was not feasible due to the nature of communicable diseases because the outcome of each patient is not independent with respect to other patients within the same hospital. Similarly, randomization at the level of hospital wards or floors was not feasible because hospital-wide changes to workflows could lead to “spillover effects” on practitioner behaviors and outcomes across units because both practitioners and patients typically moved among various locations. However, the pre-post study design has its own limitations, compounded by our relatively long implementation period (4 months). Although this allowed for a gradual rollout that helped health care staff familiarize themselves with the new workflows as the education efforts ensued, we needed to exclude data that occurred within this gap. In addition, the pre-AI period coincided with the COVID-19 pandemic, leading to substantial, systematic differences between pre-post samples in terms of patient characteristics (Table 1). Specifically, during the pre-AI period, more hospitalizations were related to COVID-19, involving patients without prior hospitalization or recorded comorbidities and resulting in a relatively low unadjusted CDI incidence rate. We adjusted for this difference using matching but cannot rule out the possibility of residual confounding. The pre-post design also means our implementation occurred within a dynamic antimicrobial stewardship environment, where other initiatives may have had overlapping effects. Although we conducted additional subanalyses to assess the robustness of our findings (eResults in Supplement 1), we cannot fully exclude the influence of concurrent interventions. Additionally, our study was not able to capture all outcomes influenced by the AI tool. For example, through anecdotal conversations, we discovered that certain nursing teams were using the CDI high-risk alerts to inform nurse-to-patient assignments, specifically, assigning the same nurse to both a CDI-positive patient and a CDI high-risk patient was avoided. Although it is encouraging to see hospital staff independently finding innovative ways to leverage our AI tool, we were not prepared to systematically measure such outcomes. Similarly, our analysis on antibiotic use focused on facility-wide global trends instead of individual changes in prescribing patterns because those were difficult to capture from our data. Future study should consider better-quality data to support such evaluations.

## Conclusions

In this quality improvement study, although the implementation of an AI-guided infection prevention bundle for CDI was not associated with a significant reduction in CDI incidence, it was associated with reductions in the use of CDI-associated antimicrobials, demonstrating the potential of AI-guided antimicrobial stewardship. The difference observed between antimicrobial stewardship and hand hygiene initiatives underscores the importance of careful implementation designs that effectively integrate AI tools into existing clinical workflows. We identified several barriers to implementation, including challenges related to technology infrastructure, staff knowledge, and acceptance of AI-guided workflows. Despite limitations of our single-center, quasi-experimental design, the insights gained from this study are broadly applicable to other institutions seeking to use AI models to improve clinical outcomes. Future research should focus on developing novel strategies of ongoing education and stakeholder engagement to overcome implementation barriers, exploring the scalability of these efforts across diverse health care settings, identifying optimal alert thresholds that balance sensitivity and alert burden, and conducting randomized clinical trials to assess their effect on clinical outcomes.

## Notes

Supplement 1. **eMethods.** Supplemental Methods **eResults.** Supplemental Results **eFigure 1.** Simulation Results of Expected Outcomes Under Various Alerting Frequencies and Different Assumed Intervention Effectiveness **eFigure 2.** Simulation Results of Model Performance Characteristics for the Selected Threshold **eFigure 3.** Overview of AI-Guided Infection Prevention Bundle **eFigure 4.** Screenshot of BPA #1 for Enhance Hand Hygiene **eFigure 5.** The “Enhance Hand Hygiene” Sign to be Posted on Patient’s Doors **eFigure 6.** Informational Flyer for Patients Receiving the Enhanced Hand Hygiene Sign **eFigure 7.** Screenshot of BPA #2 for Recommendations for Reducing CDI Risk **eFigure 8.** Mock-Up of Screenshots of Pharmacist Medication Review Interface **eFigure 9.** Cohort Inclusion and Exclusion **eFigure 10.** Descriptive Statistics of Model Implementation Assessment During the Post-AI Period **eTable 1.** 2021 Simulation Cohort Characteristics **eTable 2.** Characteristics of the Pre-Intervention and Post-Intervention Samples **eTable 3.** Primary and Secondary Outcomes for Unadjusted Analysis **eTable 4.** Denominators for Outcome Normalization **eTable 5.** Primary and Secondary Outcomes for Adjusted Analysis for Hospitalizations Eligible for the Intervention (i.e., High-Risk) **eTable 6.** Primary and Secondary Outcomes for Adjusted Analysis for Hospitalizations Ineligible for the Intervention (i.e., Low-Risk) **eTable 7.** Overall Perceptions of AI-Guided Initiative **eTable 8.** Summary Data About How Different Hospital Personnel Engaged With and Felt About Various AI-Guided Infection Prevention Bundle Components **eAppendix 1.** Semi-Structured Interview Protocols **eAppendix 2.** Field Observation Recording Templates
eReferences

Supplement 2.
Data Sharing Statement
