# Supplementary material for: Guiding Clostridioides difficile Infection Prevention Efforts in a Hospital Setting With AI
Source: JAMA Netw Open. 2025 Jun 12;8(6):e2515213. doi: 10.1001/jamanetworkopen.2025.15213 (PMC12163649; doi:10.1001/jamanetworkopen.2025.15213)
Supplement: Supplement 1. — eMethods. Supplemental Methods eResults. Supplemental Results eFigure 1. Simulation Results of Expected Outcomes Under Various Alerting Frequencies and Different Assumed Intervention Effectiveness eFigure 2. Simulation Results of Model Performance Characteristics for the Selected Threshold eFigure 3. Overview of AI-Guided Infection Prevention Bundle eFigure 4. Screenshot of BPA #1 for Enhance Hand Hygiene eFigure 5. The “Enhance Hand Hygiene” Sign to be Posted on Patient’s Doors eFigure 6. Informational Flyer for Patients Receiving the Enhanced Hand Hygiene Sign eFigure 7. Screenshot of BPA #2 for Recommendations for Reducing CDI Risk eFigure 8. Mock-Up of Screenshots of Pharmacist Medication Review Interface eFigure 9. Cohort Inclusion and Exclusion eFigure 10. Descriptive Statistics of Model Implementation Assessment During the Post-AI Period eTable 1. 2021 Simulation Cohort Characteristics eTable 2. Characteristics of the Pre-Intervention and Post-Intervention Samples eTable 3. Primary and Secondary Outcomes for Unadjusted Analysis eTable 4. Denominators for Outcome Normalization eTable 5. Primary and Secondary Outcomes for Adjusted Analysis for Hospitalizations Eligible for the Intervention (i.e., High-Risk) eTable 6. Primary and Secondary Outcomes for Adjusted Analysis for Hospitalizations Ineligible for the Intervention (i.e., Low-Risk) eTable 7. Overall Perceptions of AI-Guided Initiative eTable 8. Summary Data About How Different Hospital Personnel Engaged With and Felt About Various AI-Guided Infection Prevention Bundle Components eAppendix 1. Semi-Structured Interview Protocols eAppendix 2. Field Observation Recording Templates eReferences [file jamanetwopen-e2515213-s001.pdf]

Supplemental Online Content

Tang S, Shepard S, Clark R, et al. Guiding Clostridioides difficile infection prevention effort in a hospital setting with AI. *JAMA Netw Open*. 2025;8(6):e2515213. doi:10.1001/jamanetworkopen.2025.15213

eMethods. Supplemental methods
eResults. Supplemental results
eFigure 1. Simulation results of expected outcomes under various alerting frequencies and different assumed intervention effectiveness
eFigure 2. Simulation results of model performance characteristics for the selected threshold
eFigure 3. Overview of AI-guided infection prevention bundle
eFigure 4. Screenshot of BPA #1 for Enhance Hand Hygiene
eFigure 5. The “Enhance Hand Hygiene” sign to be posted on patient’s doors
eFigure 6. Informational flyer for patients receiving the enhanced hand hygiene sign
eFigure 7. Screenshot of BPA #2 for recommendations for reducing CDI risk
eFigure 8. Mock-up of screenshots of pharmacist medication review interface
eFigure 9. Cohort inclusion and exclusion
eFigure 10. Descriptive statistics of model implementation assessment during the post-AI period
eTable 1. 2021 simulation cohort characteristics
eTable 2. Characteristics of the pre-intervention and post-intervention samples
eTable 3. Primary and secondary outcomes for unadjusted analysis
eTable 4. Denominators for outcome normalization.
eTable 5. Primary and secondary outcomes for adjusted analysis for hospitalizations eligible for the intervention (i.e., high-risk)
eTable 6. Primary and secondary outcomes for adjusted analysis for hospitalizations ineligible for the intervention (i.e., low-risk)
eTable 7. Overall perceptions of AI-guided initiative
eTable 8. Summary data about how different hospital personnel engaged with and felt about various AI-guided infection prevention bundle components
eAppendix 1. Semi-Structured Interview Protocols
eAppendix 2. Field Observation Recording Templates
eReferences

This supplemental material has been provided by the authors to give readers additional information about their work.

© 2025 Tang S et al. *JAMA Network Open.*

eMethods

Details of AI-Guided Infection Prevention Bundle

Model Specification

The version of the model used in this study was trained on MM data collected between 2015 and 2019 and retrospectively validated on MM data from 2020, where the model was reported to achieve an area under the receiver operating characteristic curve (AUROC) of 0.801 (95% confidence interval [CI], 0.777 to 0.823).

Model Integration

After retrospective validation, the model was integrated into the EHR system at MM (Epic Systems Corporation, Verona, WI). We hosted the model on a Windows virtual machine within the hospital's secure information technology (IT) system and utilized dedicated software infrastructures to process real-time EHR data. Every day at midnight, the database job starts running and freezes the cohort data; the job queries the Epic system using real-time web services for patients EHR data and takes several hours to complete. At 6am every day prior to morning rounds (typically between 7-10am), the model calculates risk scores for all hospitalized patients who had not tested positive for *C. difficile* and were present in the hospital census at the preceding midnight database freeze. Calculated risk scores were written back to the EHR as flowsheet records. Prospective validation of the model running in "live" mode was completed in 2021 and reported in previous publications; the reported prospective AUROC was 0.767 (95% CI, 0.737 to 0.801) for a patient cohort admitted between July 2020 and June 2021.

Selection of Implementation Units and Alert Threshold

We performed a simulation analysis on a cohort of patients admitted in 2021 data. Following prior work, we extracted data for adult (≥18 years old) in-patient encounters with an admission date between Jan 1, 2021 and Dec 31, 2021, and excluded encounters that did not span at least 2 calendar days (for whom no prediction could be made). We further excluded patients with a positive *C. difficile* test in the past 14 days prior to admission. Inpatient encounters were identified as those with at least one recorded inpatient location (out of a total of 44 inpatient hospital units). For each encounter, we extracted and processed their daily EHR data and applied the model to compute a trajectory of daily risk scores. We included days until before Mar 2, 2022 (Jan 1, 2022 plus 60 days) to handle boundary cases for a more complete picture of patients admitted near the end of 2021.

Each encounter was represented by a trajectory of risk scores, one for each calendar day, starting from the day of inpatient admission until one of the following end points. If the patient had tested positive for *C. difficile*, the last day of their trajectory is the day before the day of the positive *C. difficile* test. Otherwise, the last day of the trajectory is the day before the day of inpatient discharge. In the end, this resulted in patient-days that are in-hospital and CDI-free. As a sanity check, we performed retrospective evaluation on this cohort following the same evaluation protocol as prior work, by calculating AUROC at the encounter level with the maximum risk score for each encounter. This reflects the scenario where at any particular threshold, once a patient's risk score exceeds that threshold, it is immediately acted upon. Among 39,906 encounters with a class ratio of 0.885% CDI-positives (**eTable 1**), the model achieved an AUROC of 0.822 (95% CI, 0.804 to 0.838).

From a technical standpoint, the main design decisions for the AI-guided infection prevention bundle included (i) which units to deploy the intervention, and (ii) the alert threshold. Given a set of intervention units and an alert threshold, we used the data extracted above to simulate the expected outcome rate. We assumed an intervention (with some pre-defined effectiveness level) would be applied immediately on the day when a patient's risk exceeds the alert threshold. That is, if a patient tested positive for *C. difficile* and their score exceeded the threshold on some day before the day of the positive test, we assume that an intervention was applied on that day such that they would no longer test positive later, with probability corresponding to the effectiveness level. We also assumed the outcomes of all other patients remain unchanged, i.e., no harmful effects on false positives where the intervention is applied to a patient who

© 2025 Tang S et al. *JAMA Network Open.*

originally did not test positive, and no change in outcomes for patients to whom we did not apply the intervention. In other words, we aimed to maximize the true positive rate (TPR) in the selected units.

Based on conversations between the study team and hospital leadership, we decided on a design requirement of 10 intervention units and no more than 5 alerts per unit per week on average. In the end, we sought to find an alert threshold and a set of intervention units within our design requirement that would lead to the best chance of detecting a reduction in the (expected) outcome rate.

Unfortunately, searching exhaustively on all subsets of 10 intervention units and all possible alert thresholds is prohibitively expensive: searching all combinations of 10 units out of 44 results in a combinatorically large number of over 2 billion possibilities. Therefore, we first narrowed our search space to 29 candidate units that are considered “regular” adult inpatient units, removing 9 overflow units whose functionality and staffing might change, and 6 maternity units where the patient clinical physiology is different from our target patient cohort. Note that the original list of 44 already excluded short stay and observation wards where patients are not expected to stay for a prolonged period. We then proceeded with the search in three stages. In the first stage, we assumed a fixed alert threshold of the 90th percentile of risk scores and calculated the expected reduction in outcome rates for all subsets of 20 units (out of 29), considering all 10 million possibilities and selecting the 20-unit combination that had the highest TPR. In the second stage, we repeated the same procedure to select 10 units out of the 20 from the first stage, considering 184,756 possibilities. Finally, using the selected 10 units, we varied the alert threshold and assumed effectiveness level of intervention and plotted the resulting expected outcome rates (**eFigure 1**).

Based on the simulation results, we selected an alert threshold based on the 91st percentile of the risk scores, leading to a sensitivity of 31%, positive predictive value of 4.5%, and an expected alert frequency of 5 alerts per week per hospital unit on average, and 2,600 alerts in total over the entire year (52 weeks). **eFigure 2** top panel shows where this threshold lies in relation to all possible thresholds. **eFigure 2** bottom panel shows the distribution of alert frequency over different times of the year and different days of the week.

Infection Prevention Bundle Design

When designing the study, we carefully considered the most appropriate intervention to reduce pathogen exposure and host susceptibility in line with our institutional guidance. Conversations about bundle design took place both in-person (e.g., via face-to-face meetings with physicians on the wards) and remotely (e.g., teleconferencing with pharmacists) as well as asynchronously (e.g., email exchanges of protocol drafts and interface mockups). Below, we provide motivations and justifications for each intervention.

- **Enhanced hand hygiene:** Although recent evidence might suggest gloving as an acceptable alternative to handwashing, the infection control policy at our institution did not consider gloving after hand hygiene with Purell only (without soap-and-water handwashing) to be equivalent to handwashing, given *C. difficile* spores are resistant to alcohol. Thus, our intervention recommended soap-and-water handwashing upon *room entry* to avoid bringing the pathogens to high-risk patients. Our policy did not apply *upon room exit*, which contrasts with standard diarrheal contact precautions that applies upon room exit. In addition, we also confirmed the availability of a sink for each room in the units we considered, although some units have sinks inside the room.
- **Beta-lactam allergy evaluation consults:** While piperacillin/tazobactam, amoxicillin/clavulanate, and third-generation cephalosporins are associated with higher risk for *C. difficile*, many commonly used beta-lactams such as cefazolin and amoxicillin are generally considered low risk for *C. difficile*. The goal of the beta-lactam allergy evaluation is to safely de-label inaccurate or inappropriate allergy documentation, which would allow for de-escalations from higher-risk non-beta-lactams (e.g., clindamycin or fluoroquinolones) to lower-risk beta-lactams. Based on past data collected by another study team at our institution,[1] it was reported that >90% of the patients were de-labeled following the evaluation.

- **Antimicrobial de-escalations:** Medication change recommendations of our intervention came from two possible pathways: pharmacist medication reviews, as well as manual chart review by study team physicians. Since the study concluded in Dec 2023, we have paused both the hand hygiene and study team chart review components, while the pharmacy-based medication review component remains ongoing.
    - The pharmacy-led component was integrated directly into the existing medication review dashboard. Such interventions were entered using a dropdown menu (as shown in **eFigure 8**) and recorded as Epic iVent, which included the following options: discontinued antibiotics, shortened duration of antibiotics, narrowed spectrum of antibiotics, reduced acid suppression therapy, and other. We considered the first three to be "de-escalation of antibiotics."
    - For the manual chart review, although it is quite resource intensive nature (as it required dedicated personnel), this component was intended more as a supplementary mechanism to support early implementation. The specific recommendations from chart reviews were left to the discretion of the study team member in charge on each day and were recorded as unstructured notes in a shared spreadsheet.
- **Recommending the patient eat yogurt**: We acknowledge that current evidence for yogurt or probiotics in *C. difficile* prevention remains mixed. However, one study has suggested that the Danone yogurt product used at our institution may offer potential protective benefits.[2] While we could not obtain data to measure the impact of this component, we included it as a low-risk intervention within our overall bundle and have reported it in Methods for transparency. Note that this does not apply to patients who have history of bone marrow transplant or are neutropenic.

Quasi-Experimental Analysis

Pre- and Post-AI Samples

Our study sample included adult inpatient hospitalizations (age at admission ≥18 years) from September 1, 2021 through December 31, 2023. We excluded psychiatry patients and hospitalizations shorter than 24 hours. The pre-AI period was from September 1, 2021 to August 31,2022, and the post-AI period was from January 1, 2023 to December 31, 2023. For both periods, we included complete hospitalizations that occurred within the period (i.e., admitted on or after the first day of the period and discharged on or before the last day of the period) for whom we had complete records of their entire hospitalization and whose outcomes occurred within the study periods.

Sample Characteristics

To characterize the differences in the pre- and post-AI cohorts, we first conducted a descriptive analysis of demographic and clinical characteristics which included gender, age in years, self-reported race, ethnicity, month of admission (for seasonality), unit of admission, length of stay, whether the hospitalization is COVID-related (by lab tests or by diagnosis), hospitalization history, medication exposures, and comorbidity history.

- Gender categories include women, men, unknown.
- Self-reported race and ethnicity: The EHR asks patients for race and ethnicity separately, and therefore we report them separately without remapping. The EHR allows patients to select multiple race categories from White, Black, Asian, Native Hawaiian or Pacific Islander, American Indian or Alaska Native, Patient Refused, Unknown, or missing. For our analysis, we reported the race category as White, Black, Asian, and Other (which included all patients who did not select any of White, Black, or Asian). Ethnicity categories were reported as Hispanic/Latino, Non-Hispanic/Latino, other (which included Other, Unknown, Patient Refused, or missing).
- Month of admission: we consider groupings of Jan-Apr, May-Aug, Sept-Dec.
- Unit of admission: There are 44 adult inpatient hospital units at MM. For the unit of admission, we considered the first inpatient unit that a patient arrives at - this excludes the emergency

© 2025 Tang S et al. *JAMA Network Open.*

departments, short-stay, and observation wards. The location types are mapped according to CDC NHSN instructions.[3]

- COVID-related hospitalizations: we used a composite definition that considers positive lab tests and ICD-10 diagnosis codes (to account for transfers or labs done elsewhere).
- Comorbidity history: For each hospitalization, the history of comorbidities was calculated by looking at ICD-10 diagnosis codes from all encounters with MM in the 90 days prior to the start of the index hospitalization. We consider the individual components of Charlson comorbidity index[4,5] as well as inflammatory bowel disease, a condition that is known to be associated with increased risk CDI.[6]
- Medication exposures: we specifically looked at exposure to antimicrobials and acid suppressants, according to their VA class code[7] and RxNorm ingredient names[8] which were available in the RDW database.

Statistical Analyses - Matching

To avoid leaking information about interventions or outcomes when performing matching or evaluating matching results, we focused on covariates available at the time of admission and took caution to exclude covariates that were associated with interventions (e.g., medication exposures) or those associated with outcomes (e.g., prior CDI, colonization pressure, length of stay). We performed 1:1 nearest-neighbor matching with replacement, with ties broken randomly. We repeated the matching procedure 1,000 times with several variations of distance metrics, weighting schemes, and/or included covariates, and selected the run that led to the best balance in the samples' demographic and clinical characteristics, i.e., minimized the number of differences in covariates that were significantly different. The final matching cohort was based on a weighted Mahalanobis distance that upweights 90-day hospitalization history due to its prominence in the pre-post sample differences.

Outcomes – C. difficile tests

We consider two types of laboratory tests used during the study period to identify CDI, namely the "C. difficile PCR/toxin algorithm (PCRCD)" and the "Gastrointestinal Pathogen Panel (GIPAN)".[9-11] Both tests first conduct a PCR (polymerase chain reaction) test and reflexes to a toxin test by EIA (enzyme immunoassay) only if the PCR test is positive. Therefore, there are three possible test results: PCR-, PCR+ toxin+, and PCR+ toxin-. For the primary outcome, we only count the tests that were PCR+ toxin+. For secondary outcomes, we reported the total number of tests and PCR+ results (including both toxin+ and toxin-). We also hospital-onset CDI, defined as a test result of PCR+ toxin+ on hospital-day 4 or later.[12]

Outcome – Antimicrobials and Acid Suppressants

Data for antimicrobials and acid suppressants were extracted from the inpatient medication administration records (MAR) in the EHR. We considered all medication routes and included only records that were administered, removing those recorded as ordered but not administered. For antimicrobials, we considered ampicillin/sulbactam, piperacillin/tazobactam, ceftriaxone, cefepime (concurrently with metronidazole), carbapenems (meropenem, imipenem, and ertapenem), clindamycin, fluoroquinolones (ciprofloxacin, moxifloxacin, and levofloxacin), and vancomycin. For acid suppressants, we considered proton-pump inhibitors (PPIs; omeprazole, lansoprazole, pantoprazole, and esomeprazole. and H2 blockers (famotidine, cimetidine, and nizatidine). Data for each medication were identified via a keyword search of the generic names in the medication name and order name fields in the MAR.

Outcome – Denominators and Normalization

We reported all outcomes in accordance with the guidelines published by CDC NHSN.[12-14] CDI incidence rates were reported as the number per 10,000 patient days. Antimicrobial use and acid suppressant use were reported as the days of therapy per 1,000 days present. Denominators for normalization were calculated from admission and discharge timestamps following NSHN definitions and used 12:00:00 noon as the cutoff time.

© 2025 Tang S et al. *JAMA Network Open.*

Outcome – Subgroup Analyses
To further delineate the effects arising from our interventions, we divided each of the pre-AI and post-AI samples into two subgroups: those eligible for the intervention and those ineligible. Patient hospitalizations whose daily risk scores exceeded the alert threshold were deemed eligible for the intervention. For patients that tested positive for CDI during a hospitalization, we only consider their daily risk scores before the day they tested positive.

Implementation Assessment
For BPAs, we recorded how many BPAs were triggered and the recipients' responses (accepted, dismissed, snoozed, etc.;). For pharmacy medication reviews and chart reviews, we summarized the recommended or executed medication changes using Epic iVent records (for pharmacist review) and unstructured notes from a shared spreadsheet (for chart reviews).

© 2025 Tang S et al. *JAMA Network Open.*

eResults

Study Samples

The pre-AI sample included 29,179 unique patients and 39,046 unique hospitalizations; the post-AI sample included 30,404 unique patients and 40,515 unique hospitalizations (**eFigure 11**). Sample characteristics are summarized in **eTable 2**. Compared to pre-AI, the post-AI sample had a higher percentage of hospitalized patients in the age range 66-85 (p<.001), admitted during May-August (p<0.001), and with self-reported ethnicity of "other or unknown" (p=.02). In addition, the post-AI sample had fewer patients with COVID-related hospitalizations (p<.001), but a higher percentage with recent hospitalizations (p<.001), recent antimicrobial exposure (p<.001), and recent comorbidity history (acute myocardial infarction, p=0.02; congestive heart failure, peripheral vascular disease, chronic obstructive pulmonary disease, diabetes without complications, renal disease, cancer any malignancy, all p<.001). The post-AI sample also had more admissions to the HSCT ward (p<.001), the step-down unit (p<.001), or overflow wards (p<.001). The remaining demographic and clinical characteristics considered were not statistically significantly different (**eTable 2**). Though the differences appear numerically small, they are statistically significant and many of which have known associations with CDI risk (e.g., age). After adjustment by matching (**eTable 2**), the adjusted pre-intervention sample is statistically significantly different in three comorbidities, namely mild liver disease (p<.001), diabetes with complications (p<.001), and moderate or severe liver disease (p<.001).

Implementation Assessment Results

Descriptive statistics of the implementation assessment are displayed in **eFigure 12**. During the post-AI period, a total of 248,703 CDI risk scores were generated for 42,203 hospitalizations, of which 4,086 (9.7%) hospitalizations exceeded the risk threshold and were deemed high-risk. In the subpopulation of patients who stayed in at least one of the 10 selected hospital units, a total of 109,068 CDI risk scores were generated for 12,983 hospitalizations. Of these, 2,151 (16.6%) hospitalizations exceeded the risk threshold and were deemed high-risk (an average of 4.1 alerts/unit/week). Since there are multiple providers per unit, every individual provider may only get no more than two alerts per week.

For BPAs, 1,733 and 1,664 hospitalizations triggered BPA #1 and #2 for their providers, respectively. As a result, 1,647 (76.6%) hospitalizations received an order for enhanced hand hygiene precautions, and 117 (5.4%) received an order for a BLAES consultation. In the June-July field observations, we found that when the enhanced handwashing signs were posted, adherence to the sign instructions was about 6%. In the December field observations, we found that signs were posted <40% of the time the BPA instructing their use was triggered. This suggests an overall low adherence rate for soap-and-water handwashing. Assuming the two events are independent, the overall adherence rate can be estimated to be at most 6% × 40% = 2.4%. On the other hand, the total number of BLAES orders across the hospital did not change significantly between pre and post.

For pharmacist medication reviews, there were 1,175 pharmacy-based interventions using the Epic iVent tool during the post-AI period, where 37 pharmacists documented medication reviews for patients who had a high-CDI-risk flag. In 133 (12.4%) instances the pharmacist performed changes to the medications, 109 (82.0%) involved antibiotic de-escalation (discontinued, shortened duration, reduced dose intensity, or narrowed spectrum) and 22 (16.5%) reduced acid suppression therapy.

For the 2,151 high-risk hospitalizations in the 10 selected units, four ID physicians recorded 119 instances of chart reviews where they suggested an intervention, among which 109 (91.6%) messaged a first-contact provider, 11 (9.2%) messaged an ID fellow, 62 (52.1%) suggested changing piperacillin/tazobactam to cefepime/metronidazole, and 52 (43.7%) suggested de-escalating PPIs or changing to H2 blockers as an alternative agent.

For the semi-structured interviews, we recruited a total of 17 first-contact providers and 7 pharmacists (**eTable 7**). While all interviewed pharmacists were aware of this initiative, only 10/17 of interviewed

primary inpatient providers were aware. When asked about the impacts on role-specific clinical workflows, all 7/7 interviewed pharmacists performed the medication and chart reviews as prompted by our initiative. However, only 7/17 of interviewed primary inpatient providers paid attention to the BPAs, even though 14/17 of them responded positively when asked about their attitude towards these workflow changes. **eTable 8** further summarizes the key themes from the interviews. Selected quotes from providers about different bundle components they interacted with are shown in **Table 3** of main text.

Considerations for Overlapping Stewardship Initiatives

We are aware of two stewardship initiatives that occurred during our post-AI period (2023 January-December) and may have overlapped with our intervention.

- **Ventilator-associated pneumonia (VAP)**. In July 2023, pneumonia treatment guidelines[15] were updated to recommend cefepime over piperacillin/tazobactam for VAP in the ICU. To assess the potential impact of this change, we performed a sub-analysis limited to data from January-June 2023 (before the update) as the post-AI sample. The result below showed that trends in antimicrobial use, particularly for piperacillin/tazobactam and cefepime/metronidazole, were consistent with our overall findings, suggesting that the observed reductions are unlikely to be fully explained by the VAP guideline change alone.

**Outcome**	**Pre-AI (adjusted)**	**2023-01 to 2023-06**	**Change (adjusted)**	**P value (adjusted)**
Antibiotic use, days of therapy per 1,000 days present				
Piperacillin/tazobactam	59.21 (56.65, 61.59)	52.23 (49.01, 55.79)	-6.98 (-11.08, -2.55)	<.001
Cefepime, with concurrent metronidazole	9.00 (8.18, 9.89)	11.02 (9.47, 12.64)	2.01 (0.13, 3.80)	.04

- **Beta-lactam allergy BPA**. In March 2023, a separate quality improvement initiative implemented a BPA to prompt consultation with the beta-lactam allergy evaluation team for patients with beta-lactam allergy label but evidence of prior tolerance. This BPA is intended to encourage allergy re-evaluation and responding to it was voluntary. While this could have downstream influence on antibiotic selection, it was not directly tied to our intervention and likely had minimal short-term impact on prescribing patterns relevant to CDI risk. Nonetheless, we acknowledge its presence as part of the complex, dynamic stewardship environment during our study period.

© 2025 Tang S et al. *JAMA Network Open.*

eFigure 1. Simulation results of expected outcomes under various alerting frequencies and different assumed intervention effectiveness.

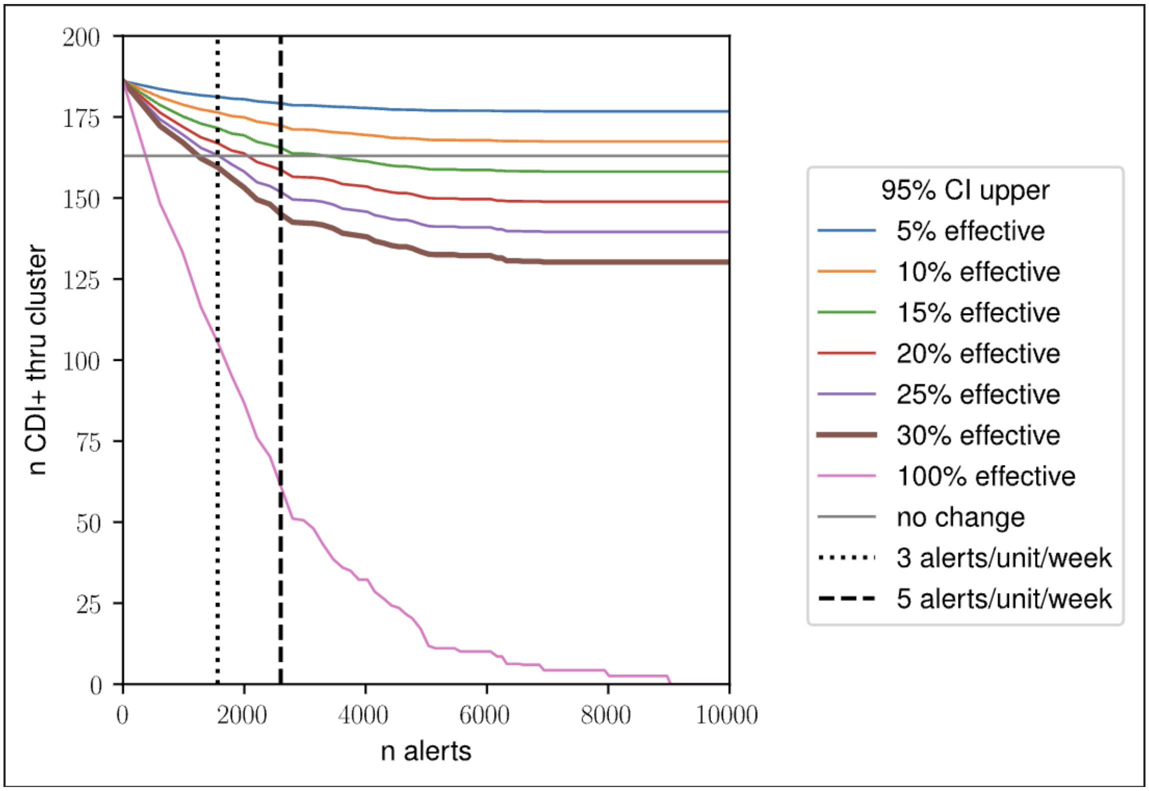

Simulation results of the expected outcome for the final selected 10 units, with varying levels of assumed effectiveness of intervention. The plotted lines are the upper bounds of the 95% CI of outcome counts based on 1,000 Monte-Carlo samples from the simulation.

© 2025 Tang S et al. *JAMA Network Open.*

eFigure 2. Simulation results of model performance characteristics for the selected threshold.

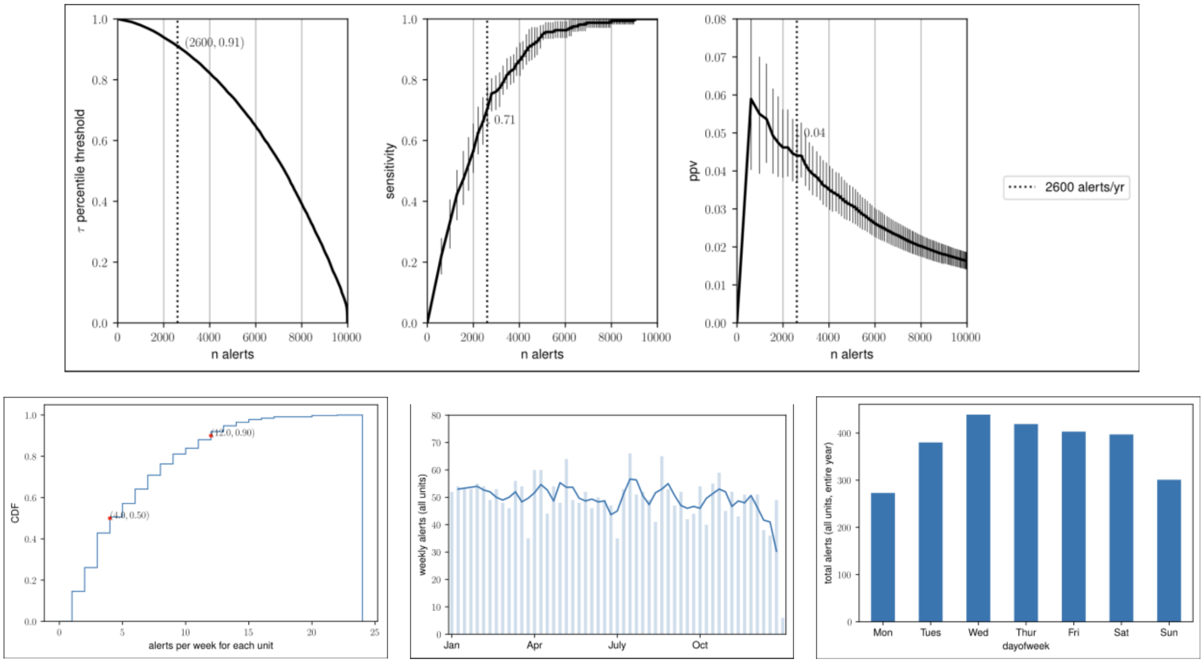

Left: Cumulative distribution function of the number of weekly alerts. While we aimed for 5 alerts/unit/week on average, not every week in the simulation had exactly 5 alerts. We expected 50% of the weeks to have no more than 4 alerts, and 90% of the weeks have no more than 12 alerts. Middle: Number of weekly alerts over the 52 weeks of the year. Right: Average number of daily alerts on different days of the week.

© 2025 Tang S et al. *JAMA Network Open.*

eFigure 3. Overview of AI-guided infection prevention bundle.

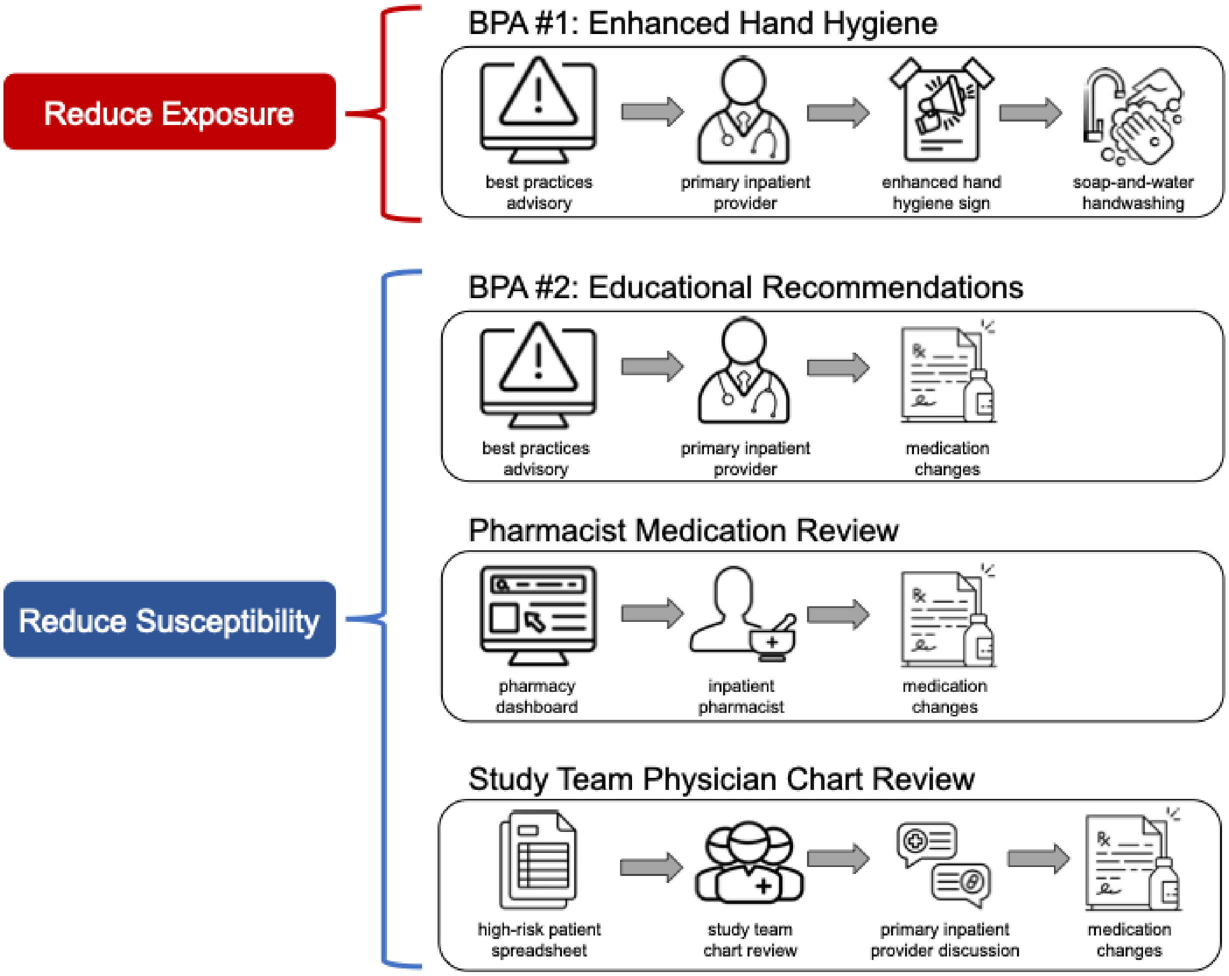

© 2025 Tang S et al. *JAMA Network Open.*

eFigure 4. Screenshot of BPA #1 for Enhance Hand Hygiene.

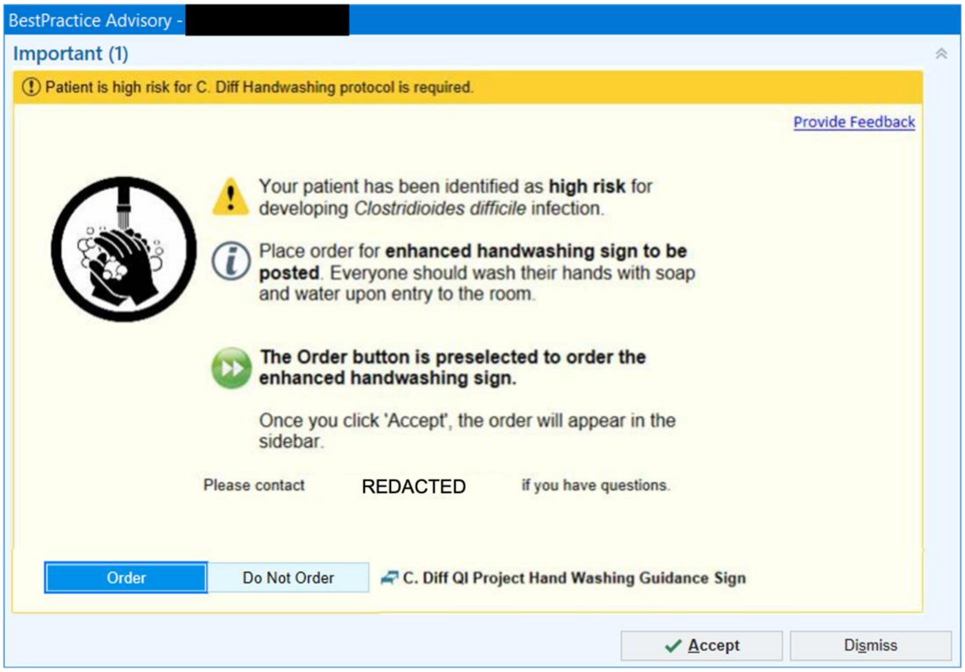

© 2025 Tang S et al. *JAMA Network Open.*

eFigure 5. The “Enhance Hand Hygiene” sign to be posted on patient’s doors.

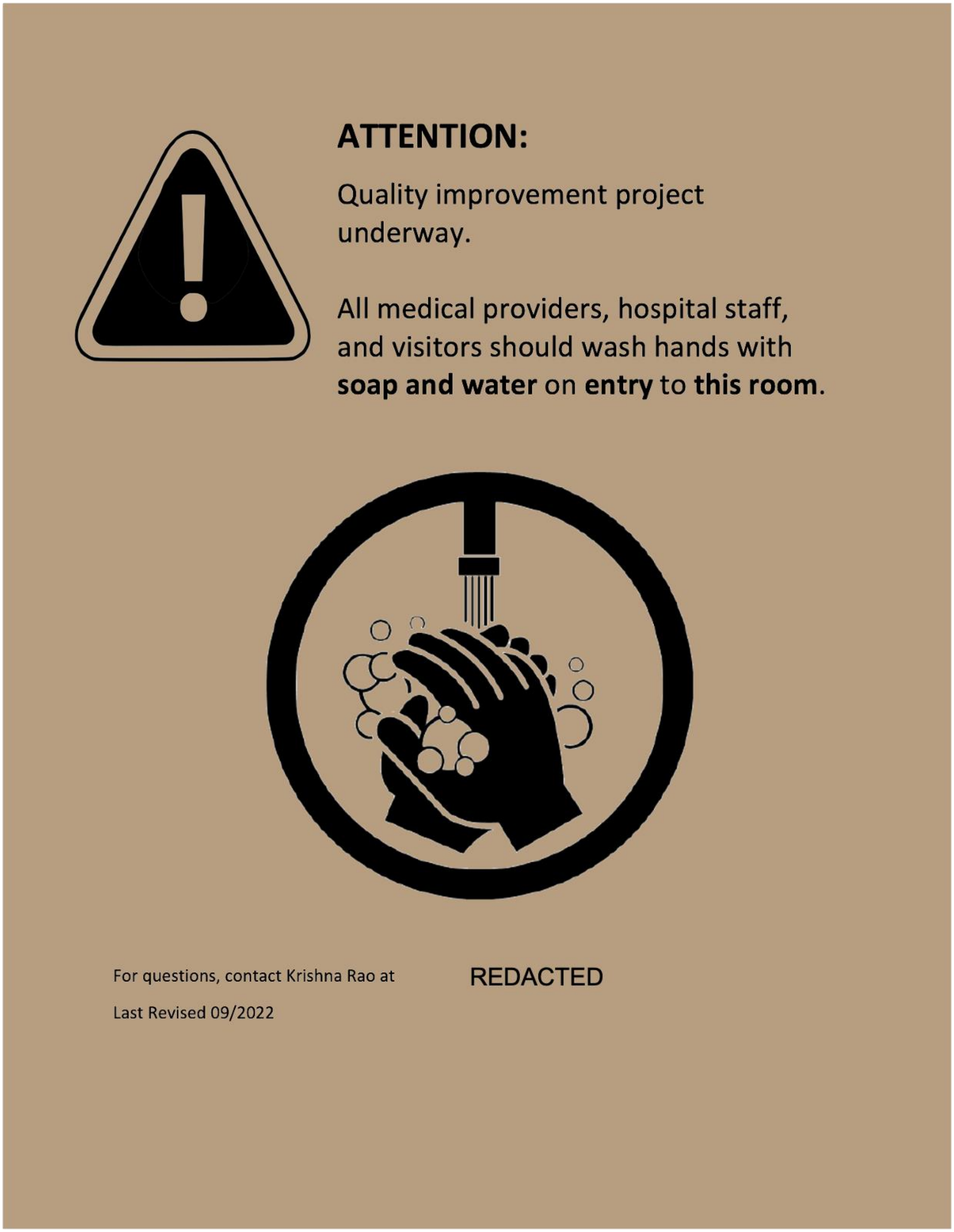

© 2025 Tang S et al. *JAMA Network Open.*

eFigure 6. Informational flyer for patients receiving the enhanced hand hygiene sign.

UNIVERSITY OF MICHIGAN HEALTH
MICHIGAN MEDICINE

(Quality Improvement Intervention) Preventing *Clostridioides difficile* Infection

Why am I receiving this handout?

Our team is exploring ways for reducing *Clostridioides difficile* infection (CDI) at the hospital. CDI is a bacterial infection that causes severe diarrhea and inflammation of the colon.

What does the sign on my door say?

The sign asks that visitors, including family members and your healthcare team, **wash their hands with soap and water** when they **enter your hospital room.**

While you **do not have an infection** with *C.difficile*, you may benefit from providers/visitors practicing hand hygiene with soap and water instead of or in addition to hand sanitizer.

This project was developed at the University of Michigan with the goal of reducing the rate of CDI at the hospital. If you have any questions, please contact REDACTED .

UNIVERSITY OF MICHIGAN HEALTH
MICHIGAN MEDICINE

- 1 -

© 2025 Tang S et al. *JAMA Network Open.*

eFigure 7. Screenshot of BPA #2 for recommendations for reducing CDI risk.

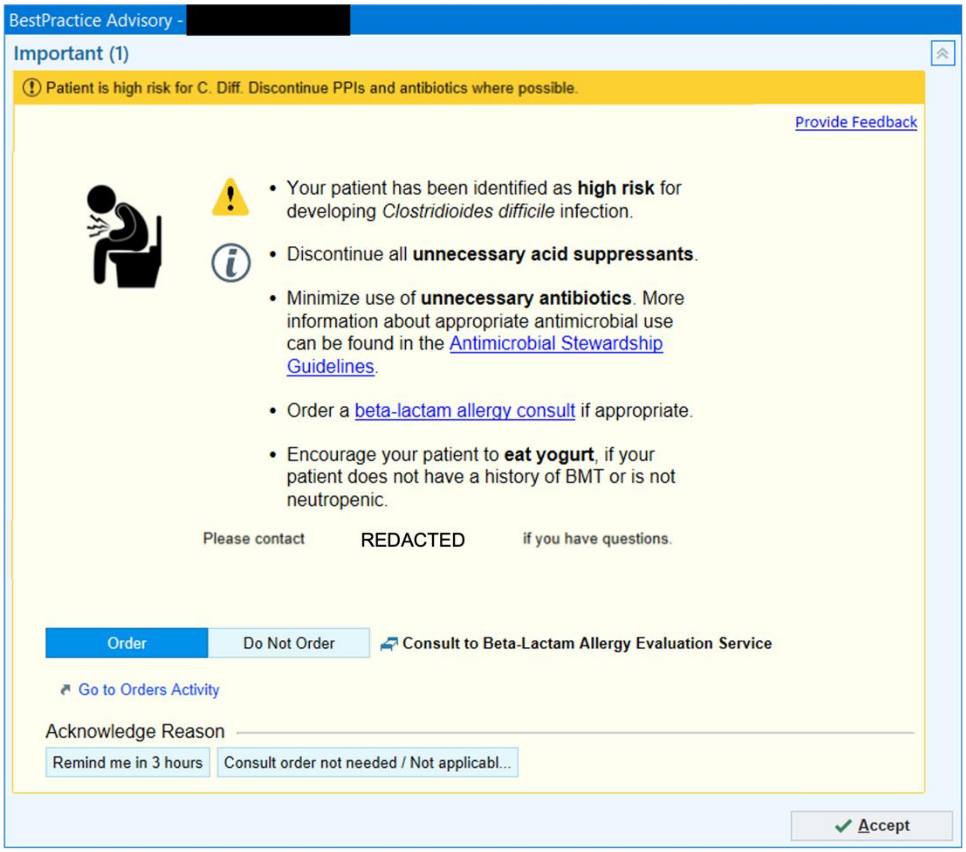

© 2025 Tang S et al. *JAMA Network Open.*

eFigure 8. Mock-up of screenshots of pharmacist medication review interface.

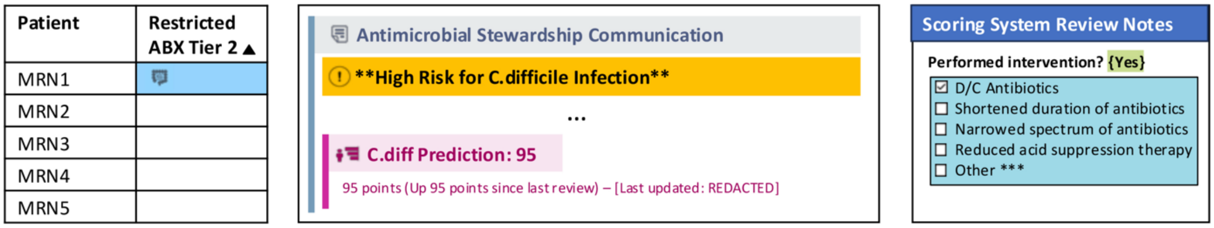

Left: in the "Restricted ABX Tier 2" column, patients with CDI risk exceeding the threshold are shown with a new icon whose shape resembles the colon. Middle: patient's chart showing information about the CDI risk. Right: pharmacists can record their intervention in response to the high-CDI-risk icon.

eFigure 9. Cohort inclusion and exclusion.

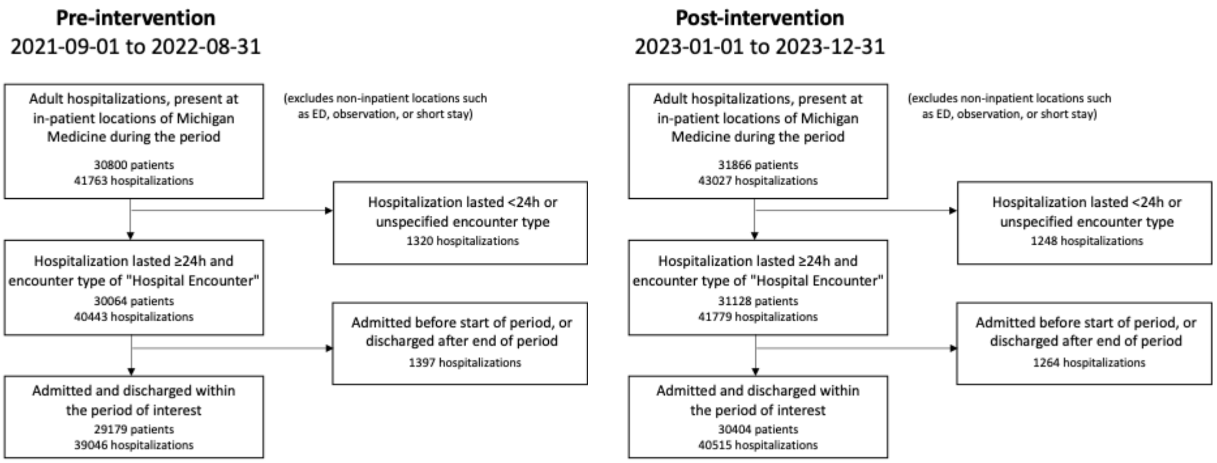

eFigure 10. Descriptive statistics of model implementation assessment during the post-AI period.

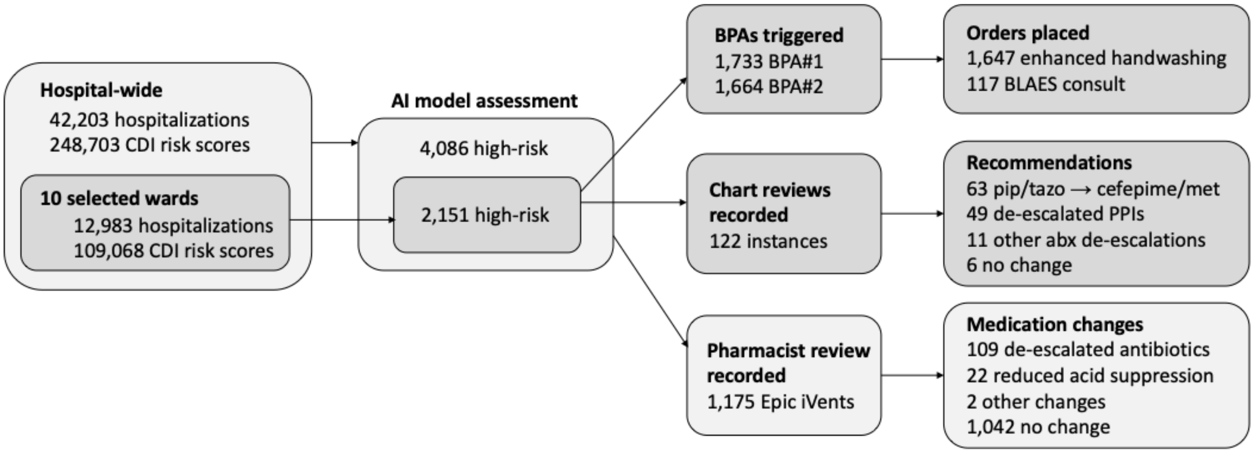

CDI, *C. difficile* infection; AI, artificial intelligence; BPAs, best practices advisory; BLAES, beta-lactam allergy evaluation service; pip/tazo, piperacillin/tazobactam; met, metronidazole; PPIs, proton-pump inhibitors.

© 2025 Tang S et al. *JAMA Network Open.*

eTable 1. 2021 simulation cohort characteristics.

Year	2021
N encounters	39,906
CDI+	353
%CDI+	0.885%
Test AUROC (95% CI)	0.822 (0.804, 0.838)
Length of stay (median IQR)	3 [1-6]
Gender	
Women	22038 55.2%
Men	17867 44.8%
Unknown	1 0%
Age at admission (median IQR)	57 [36-70]
Age at admission (min max)	18-89
Age at admission (mean std)	54.3 ± 19.2
Age at admission	
(18, 25]	2693 6.7%
(25, 45]	10900 27.3%
(45, 65]	12473 31.3%
(65, 85]	12137 30.4%
>85	1358 3.4%
Race	
Caucasian	31287 78.4%
African American	5197 13.0%
Other	1376 3.4%
Asian	1109 2.8%
Unknown	258 0.7%
American Indian or Alaska Native	262 0.6%
Patient Refused	156 0.4%
Native Hawaiian and Other Pacific Islander	43 0.1%

© 2025 Tang S et al. *JAMA Network Open.*

eTable 2. Characteristics of the pre-intervention and post-intervention samples.

Characteristic	Unadjusted Pre-AI	Adjusted Pre-AI	Post-AI	Unadjusted *P*	Adjusted *P*
Number of hospitalizations	39045	40515	40515	NA	NA
Number of unique hospitalizations	39045	23190	40515		
Number of unique patients	29179	18760	30404		
Sex				>.99	>.99
Female	21,645 55.4%	22,505 55.5%	22,575 55.7%	>.99	>.99
Male	17,399 44.6%	18,009 44.5%	17,939 44.3%	>.99	>.99
Unknown	1 <0.1%	1 <0.1%	1 <0.1%	>.99	>.99
Age at admission, median (IQR), years	58 (36-70)	58 (37-71)	58 (37-70)	<.001	>.99
Age at admission, binned				<.001	>.99
18-25	2,963 7.6%	2,824 7.0%	2,853 7.0%	.19	>.99
26-45	10,958 28.1%	10,988 27.1%	11,032 27.2%	.53	>.99
46-65	11,808 30.2%	12,306 30.4%	12,222 30.2%	>.99	>.99
66-85	11,933 30.6%	13,032 32.2%	13,019 32.1%	<.001	>.99
>85	1,384 3.5%	1,365 3.4%	1,389 3.4%	>.99	>.99
Race				.62	>.99
Asian	1,192 3.1%	1,244 3.1%	1,262 3.1%	>.99	>.99
Black	5,330 13.7%	5,096 12.6%	5,219 12.9%	.09	>.99
White	30,753 78.8%	32,342 79.8%	32,231 79.6%	.37	>.99
Other or unknown	2,034 5.2%	2,082 5.1%	2,105 5.2%	>.99	>.99
Ethnicity				.04	>.99
Non-Hispanic/Latino	36,815 94.3%	37,998 93.8%	37,968 93.7%	.04	>.99
Hispanic/Latino	1,374 3.5%	1,477 3.6%	1,503 3.7%	>.99	>.99
Other or unknown	857 2.2%	1,040 2.6%	1,044 2.6%	.03	>.99
Month of admission				<.001	>.99
January - April	13,063 33.5%	12,967 32.0%	13,117 32.4%	.07	>.99
May - August	12,896 33.0%	14,292 35.3%	14,166 35.0%	<.001	>.99
September - December	13,087 33.5%	13,256 32.7%	13,232 32.7%	.63	>.99
Length of stay, median (IQR), days	3 (2-7)	3 (2-7)	3 (2-7)	>.99	>.99
COVID-associated hospitalization				NA	NA
Positive lab	1,888 4.8%	622 1.5%	623 1.5%	<.001	>.99
Diagnosis	2,258 5.8%	879 2.2%	882 2.2%	<.001	>.99
Positive lab and diagnosis	1,629 4.2%	529 1.3%	530 1.3%	<.001	>.99
Positive lab or diagnosis	2,517 6.4%	972 2.4%	975 2.4%	<.001	>.99
Recent hospitalization	10,204 26.1%	13,550 33.0%	13,652 33.7%	<.001	>.99
Recent medication exposure				NA	NA
Antimicrobials	6,176 15.8%	7,975 19.7%	7,082 17.5%	<.001	<.001
Acid suppressants	5,483 14.0%	6,906 17.0%	5,673 14.0%	>.99	<.001
Recent comorbidity history				NA	NA
Acute myocardial infarction	1,031 2.6%	1,280 3.2%	1,238 3.1%	.03	>.99
Congestive heart failure	2,440 6.2%	3,189 7.9%	2,946 7.3%	<.001	.08
Peripheral vascular disease	1,720 4.4%	2,335 5.8%	2,182 5.4%	<.001	>.99
Cerebrovascular disease	885 2.3%	1,110 2.7%	960 2.4%	>.99	.05
Dementia	258 0.7%	268 0.7%	253 0.6%	>.99	>.99
Chronic obstructive pulmonary disease	2,291 5.9%	3,038 7.5%	3,222 8.0%	<.001	.96
Rheumatoid disease	494 1.3%	612 1.5%	566 1.4%	>.99	>.99
Peptic ulcer disease	218 0.6%	283 0.7%	251 0.6%	>.99	>.99
Mild liver disease	1,345 3.4%	1,814 4.5%	1,416 3.5%	>.99	<.001
Diabetes without complications	2,302 5.9%	2,997 7.4%	2,880 7.1%	<.001	>.99
Diabetes with complications	1,900 4.9%	2,404 5.9%	2,017 5.0%	>.99	<.001
Hemiplegia or paraplegia	455 1.2%	530 1.3%	545 1.3%	>.99	>.99
Renal disease	2,711 6.9%	3,495 8.6%	3,378 8.3%	<.001	>.99
Cancer any malignancy	2,749 7.0%	3,353 8.3%	3,485 8.6%	<.001	>.99
Moderate or severe liver disease	601 1.5%	808 2.0%	535 1.3%	.58	<.001
Metastatic solid tumor	1,116 2.9%	1,410 3.5%	1,311 3.2%	.12	>.99
AIDS/HIV	51 <0.1%	39 <0.1%	41 <0.1%	>.99	>.99
Inflammatory bowel disease	326 0.8%	463 1.1%	402 1.0%	>.99	>.99
Admission location type				<.001	>.99
Medical ICUs	1,060 2.7%	1,072 2.6%	1,072 2.6%	>.99	>.99
Surgical ICUs	2,973 7.6%	3,201 7.9%	3,201 7.9%	>.99	>.99
Step-down units	410 1.1%	639 1.6%	639 1.6%	<.001	>.99
Medical wards	12,557 32.2%	12,647 31.2%	12,644 31.2%	.24	>.99
Medical-surgical wards	4,002 10.2%	4,169 10.3%	4,171 10.3%	>.99	>.99
Surgical wards	7,729 19.8%	8,366 20.6%	8,375 20.7%	.13	>.99
Oncology wards	2,056 5.3%	2,067 5.1%	2,079 5.1%	>.99	>.99
HSCT wards	1,387 3.6%	1,162 2.9%	1,162 2.9%	<.001	>.99
Maternity wards	5,760 14.8%	5,664 14.0%	5,644 13.9%	.06	>.99
Overflow wards	396 1.0%	820 2.0%	820 2.0%	<.001	>.99
Other wards	716 1.8%	708 1.7%	708 1.7%	>.99	>.99

© 2025 Tang S et al. *JAMA Network Open.*

P-values are from z-tests for comparing proportions of binary variables and each category of categorical variables, chi-squared tests for homogeneity for comparing categorical variables, and Mann-Whitney U tests for comparing continuous variables, with a Bonferroni correction (separately for unadjusted and adjusted comparison) to adjust for multiple hypotheses (number of tests: 62).

© 2025 Tang S et al. *JAMA Network Open.*

eTable 3. Primary and secondary outcomes for unadjusted and adjusted analysis.

Outcome	Pre-AI (unadjusted)	Pre-AI (adjusted)	Post-AI	Change (unadjusted)	Change (adjusted)	P value (unadjusted)	P value (adjusted)
**Primary outcome**							
Clostridioides difficile infections [a], incidence rate per 10,000 patient days	4.98 (4.16, 5.83)	5.76 (4.87, 6.69)	5.65 (4.78, 6.56)	1.00 (-1.27, 3.26)	-0.11 (-1.43, 1.18)	.39	.85
**Secondary outcomes**							
Laboratory testing of CDI, instances per 10,000 patient days							
Test conducted	115.64 (111.65, 119.34)	119.89 (116.17, 123.73)	115.05 (111.36, 118.92)	-0.59 (-5.85, 4.93)	-4.84 (-10.16, 0.81)	>.99	>.99
PCR-positive *C. difficile* test [b]	17.10 (15.54, 18.72)	18.61 (17.04, 20.24)	18.10 (16.49, 19.75)	0.67 (-0.62, 1.90)	-0.51 (-2.89, 1.75)	>.99	>.99
Hospital-onset CDI [c]	3.49 (2.81, 4.24)	3.98 (3.24, 4.78)	3.74 (2.99, 4.54)	0.25 (-0.86, 1.24)	-0.24 (-1.36, 0.83)	>.99	>.99
Antibiotic use, days of therapy per 1,000 days present							
Ampicillin/sulbactam	18.69 (17.47, 20.03)	20.45 (19.22, 21.73)	17.63 (16.42, 18.87)	-1.07 (-2.91, 0.64)	-2.82 (-4.59, -1.03)	>.99	.03
Piperacillin/tazobactam	59.65 (57.01, 62.43)	59.21 (56.76, 61.50)	49.58 (47.28, 52.00)	-10.07 (-13.58, -6.65)	-9.64 (-12.93, -6.28)	<.001	<.001
Ceftriaxone	19.43 (17.76, 21.09)	19.20 (17.61, 20.84)	18.01 (16.65, 19.41)	-1.42 (-3.45, 0.92)	-1.19 (-3.28, 1.03)	>.99	>.99
Cefepime	39.10 (37.08, 41.26)	41.49 (39.31, 43.77)	44.07 (41.83, 46.38)	4.97 (1.75, 8.25)	2.58 (-0.74, 5.75)	.10	>.99
Cefepime, with concurrent metronidazole	9.01 (8.10, 9.97)	9.00 (8.16, 9.94)	10.98 (9.82, 12.15)	1.97 (0.47, 3.38)	1.98 (0.52, 3.43)	.20	.24
Cefepime, without concurrent metronidazole	30.09 (28.34, 31.98)	32.49 (30.63, 34.44)	33.08 (31.14, 34.85)	3.00 (0.41, 5.55)	0.60 (-2.07, 3.09)	.44	>.99
Carbapenems [d]	16.36 (14.70, 18.06)	16.05 (14.48, 17.73)	16.90 (15.27, 18.56)	0.54 (-1.92, 2.81)	0.85 (-1.43, 3.13)	>.99	>.99
Clindamycin	3.50 (3.04, 3.99)	3.38 (2.97, 3.85)	2.34 (1.98, 2.73)	-1.16 (-1.77, -0.56)	-1.04 (-1.60, -0.47)	<.001	.03
Fluoroquinolones [e]	26.08 (24.30, 27.97)	26.44 (24.57, 28.43)	28.32 (26.44, 30.17)	2.25 (-0.60, 4.86)	1.88 (-0.90, 4.43)	>.99	>.99
Vancomycin	75.95 (72.89, 79.07)	78.00 (75.15, 80.74)	67.16 (64.69, 69.86)	-8.79 (-12.78, -4.61)	-10.85 (-14.54, -6.66)	<.001	<.001
Vancomycin, oral	16.05 (14.08, 18.34)	18.25 (16.16, 20.36)	16.54 (14.79, 18.42)	0.49 (-2.33, 3.08)	-1.71 (-4.37, 1.21)	>.99	>.99
Vancomycin, intravenous	61.61 (59.13, 63.95)	61.64 (59.54, 63.87)	52.22 (50.27, 54.27)	-9.39 (-12.57, -6.29)	-9.42 (-12.37, -6.46)	<.001	<.001
Acid suppressant use, days of therapy per 1,000 days present							
proton-pump inhibitors [f]	251.94 (245.42, 257.96)	255.69 (250.21, 261.71)	249.94 (244.20, 255.43)	-1.99 (-10.87, 6.15)	-5.74 (-14.36, 2.28)	>.99	>.99
H2 blockers [g]	116.61 (111.86, 121.56)	114.49 (109.95, 118.91)	110.49 (106.12, 114.83)	-6.12 (-12.67, 0.30)	-4.01 (-10.24, 2.58)	>.99	>.99

Parenthesized ranges are 95% confidence intervals.
P-values for secondary outcomes are for two-sided tests and corrected with a Bonferroni adjustment (separately for unadjusted and adjusted analysis; number of tests: 17).
[a] Laboratory-identified CDI.
[b] Includes all tests that are positive for PCR regardless of EIA result (could be positive or negative).
[c] Subset of laboratory-identified CDI that are hospital-onset.
[d] Carbapenems include: meropenem, imipenem, ertapenem.
[e] Fluoroquinolones include ciprofloxacin, moxifloxacin, and levofloxacin.
[f] Proton-pump inhibitors include omeprazole, lansoprazole, pantoprazole, and esomeprazole.
[g] H2 blockers include famotidine, cimetidine, and nizatidine.

© 2025 Tang S et al. *JAMA Network Open.*

eTable 4. Denominators for outcome normalization.

Cohort	Unadjusted Pre-AI	Adjusted Pre-AI	Post-AI
**Denominators** (NSHN definitions)			
Patient admissions	39046	40515	40515
Patient days	255017	263825	261892
Days present	280939	291022	289417

© 2025 Tang S et al. *JAMA Network Open.*

eTable 5. Primary and secondary outcomes for adjusted analysis for hospitalizations eligible for the intervention (i.e., high-risk).

Outcome	Pre-AI (adjusted)	Post-AI	Change	p-value
**Primary Outcome**				
Clostridioides difficile infections incidence rate per 10,000 patient days	11.77 (9.29, 14.30)	10.91 (8.26, 13.50)	-0.86 (-4.35, 2.70)	>.99
**Secondary outcomes**				
Laboratory testing of CDI (count per 10,000 patient days)				
Test conducted	212.85 (203.98, 222.14)	203.11 (194.00, 212.05)	-9.74 (-22.71, 2.99)	>.99
PCR-positive *C. difficile* test	34.76 (30.46, 38.74)	32.90 (28.78, 37.34)	-1.86 (-7.95, 4.28)	>.99
Hospital-onset CDI	8.59 (6.55, 10.64)	7.33 (5.20, 9.51)	-1.26 (-4.12, 1.54)	>.99
Antibiotic use (days per 1,000 days present)				
Ampicillin/sulbactam	23.12 (20.35, 26.08)	20.52 (17.60, 23.61)	-2.60 (-6.63, 1.64)	>.99
Piperacillin/tazobactam	111.51 (104.88, 117.78)	92.73 (85.87, 100.27)	-18.78 (-28.39, -8.57)	<.001
Ceftriaxone	31.43 (27.52, 35.59)	28.01 (24.56, 31.69)	-3.43 (-8.91, 1.93)	>.99
Cefepime, concurrent with metronidazole	21.01 (18.35, 23.87)	24.39 (20.87, 28.20)	3.38 (-1.33, 8.01)	>.99
Carbapenems	39.92 (35.03, 45.05)	39.16 (34.37, 44.39)	-0.76 (-8.10, 6.66)	>.99
Clindamycin	4.52 (3.62, 5.52)	2.81 (1.88, 3.90)	-1.71 (-3.13, -0.36)	.36
Fluoroquinolones	54.96 (49.26, 60.52)	64.98 (58.69, 71.01)	10.02 (1.18, 18.40)	.36
Vancomycin	148.85 (140.74, 157.03)	123.76 (116.03, 131.59)	-25.08 (-35.95, -13.43)	<.001
Acid suppressant use (days per 1,000 days present)				
proton-pump inhibitors	353.26 (339.67, 368.30)	323.36 (307.86, 339.13)	-29.91 (-50.20, -8.86)	.12
H2 blockers	172.94 (161.42, 184.41)	170.20 (156.92, 183.42)	-2.75 (-20.07, 16.32)	>.99

Footnotes: see eTable 3.

© 2025 Tang S et al. *JAMA Network Open.*

eTable 6. Primary and secondary outcomes for adjusted analysis for hospitalizations ineligible for the intervention (i.e., low-risk).

Outcome	Pre-AI (adjusted)	Post-AI	Change	p-value
**Primary Outcome**				
Clostridioides difficile infections [1] incidence rate per 10,000 patient days	3.50 (2.68, 4.36)	4.04 (3.15, 4.94)	0.54 (-0.60, 1.85)	>.99
**Secondary outcomes**				
Laboratory testing of CDI (count per 10,000 patient days)				
Test conducted	84.86 (80.89, 88.63)	88.08 (84.35, 91.86)	3.22 (-2.26, 9.11)	>.99
PCR-positive *C. difficile* test	12.53 (10.95, 14.13)	13.57 (11.92, 15.14)	1.04 (-1.27, 3.41)	>.99
Hospital-onset CDI	2.24 (1.56, 2.91)	2.64 (1.98, 3.38)	0.40 (-0.50, 1.42)	>.99
Antibiotic use (days per 1,000 days present)				
Ampicillin/sulbactam	19.49 (18.03, 20.95)	16.78 (15.47, 18.04)	-2.71 (-4.60, -0.82)	.17
Piperacillin/tazobactam	40.49 (38.46, 42.69)	36.96 (34.94, 38.96)	-3.52 (-6.49, -0.67)	.28
Ceftriaxone	14.83 (13.31, 16.34)	15.09 (13.67, 16.61)	0.27 (-1.77, 2.31)	>.99
Cefepime, concurrent with metronidazole	4.70 (4.03, 5.43)	7.07 (6.09, 8.06)	2.36 (1.09, 3.63)	<.001
Carbapenems	7.51 (6.57, 8.67)	10.39 (9.10, 11.81)	2.89 (1.22, 4.64)	.03
Clindamycin	2.97 (2.58, 3.48)	2.20 (1.85, 2.60)	-0.77 (-1.37, -0.18)	.22
Fluoroquinolones	16.23 (14.72, 17.85)	17.61 (16.09, 19.16)	1.37 (-0.92, 3.43)	>.99
Vancomycin	52.63 (50.18, 54.94)	50.61 (47.88, 53.05)	-2.02 (-5.32, 1.14)	>.99
Acid suppressant use (days per 1,000 days present)				
proton-pump inhibitors	220.75 (214.90, 226.29)	228.49 (222.75, 233.95)	7.74 (-0.13, 15.71)	.73
H2 blockers	93.56 (89.39, 98.22)	93.04 (88.99, 97.05)	-0.52 (-6.50, 5.53)	>.99

Footnotes: see eTable 3.

© 2025 Tang S et al. *JAMA Network Open.*

eTable 7. Overall perceptions of AI-guided initiative.

	Primary Inpatient Provider (n=17)	Inpatient Pharmacist (n=7)
Aware of the initiative	10/17	7/7
Workflow impacts		
- Do you like the idea?	14/17	5/7
- Did you do what was asked (e.g., review charts, pay attention to BPAs)?	7/17	7/7
Should the initiative continue?		
- Yes, it's helpful/important	7/17	3/7
- Neutral	9/17	2/7
- Mixed, some parts are good and some may be less helpful	0/17	0/7
- No, it isn't helpful/we already do these things	1/17	2/7

© 2025 Tang S et al. *JAMA Network Open.*

eTable 8. Summary data about how different hospital personnel engaged with and felt about various AI-guided infection prevention bundle components.

Component	Role	Themes	Ratio
**Reduce Exposure**			
Best Practice Alerts	First Contacts	Remembers BPAs	7/17
		Ignores or Dismisses BPAs	6/17
Enhanced Handwashing	First Contacts	Barrier – Time	11/17
		Barrier – Sink Availability/Location	9/17
**Reduce Susceptibility**			
Medication reviews	Pharmacists	Fits into workflow	6/7
		Extra documentation was disruptive	1/7
Pharmacist and Care Team Communication	First Contacts	Was contacted by a Pharmacist/ID Physician	6/17
		Positive perception	4/6
	Pharmacists	Communicated during team rounds	6/7
		Communicated using secure chat	5/7
		Positive perception	4/7
Reduce or Discontinue Antibiotics or PPIs	First Contacts	Frequently complied with recommendations	2/6
		Interventions weren't appropriate for patients	4/17
		Wanted more details on model features	0/17
	Pharmacists	Care team frequently complied with recommendations	6/7
		No antimicrobial intervention possible	4/7
		Wanted more details on model features	4/7
Recommend Yogurt	First Contacts	Recommended Yogurt	6/17
		Recommended Kefir or Fermented Foods	2/17
		Did not recommend yogurt	11/17
Beta-Lactam Allergy Consult	First Contacts	Did not place an order	16/17

© 2025 Tang S et al. *JAMA Network Open.*

eAppendix 1. Semi-Structured Interview Protocols.

Physician Interview Guide – CDI risk prediction

Introduction / Consent
Hi, my name is [interviewer name] and I'll be leading the interview. Thank you so much for sharing your time with us today. Before we begin, I have some consent information to read through and then I'll give you the chance to ask any questions you might have about the study or the consent process.
[Review Information Sheet]
Do you have any questions for us about the interview or the consent process?
Okay, I am going to turn the recorder on now and ask for your consent so that we have it on record.
TURN RECORDER ON
For the record, do we have your permission to conduct and audio-record this interview?
[If YES – continue to questions]
[If NO – stop recorder and ask if it is okay to complete the interview without recording if study team only takes notes. If subject agrees, continue to questions and make note that interview was not audio recorded per subject request.]

Interview Questions
Our goal in this interview is to learn more about your experience with an initiative to reduce the risk of C difficile infections in patients who are at high risk. A team at Michigan Medicine has developed a prediction model to identify patients who are at high risk, and for those patients there are several strategies we that have deployed to help reduce their risk. One piece you may be familiar with is a quality improvement project sign, printed on brown or white paper, that encourages handwashing with soap and water on room entry. In addition, there is an automated medication review performed by a pharmacist or ID physician to ensure that patients are on appropriate antibiotics, clinicians are advised to talk with their patients about eating yogurt and can order a beta-lactam allergy consult. These interventions are being implemented for patients on several units in the hospital.

1. To get started, could you tell us a bit about your clinical duties in the hospital?
    a. How long have you worked there?
    b. How many patients do you have at a time in [names of intervention units]??
    c. What is the patient acuity?

2. How familiar are you with this C difficile prevention program?
    a. How often do you receive alerts related to a patient at high risk for C. diff?
    b. As there is more than one alert, do you recall what's in the alerts that you have received? [order enhanced hand hygiene precautions, discuss yogurt with patient, order beta-lactam consult, discontinue unnecessary acid suppressants, minimize use of unnecessary antibiotics]
    c. Please describe how you generally proceed after receiving the alert.

3. How often do you have patients who have the quality improvement handwashing (Brown/White sign) sign posted?
    a. What are the biggest challenges with handwashing on room entry?
    b. How easy or difficult is it to wash your hands with soap and water when entering the room?
    c. What do you think would make it easier to wash your hands for patients with a sign posted?
    d. Did the handwashing signs change how you went about your work in any way? Why/why not.
    e. Did you ever go into the room less often than you might have otherwise?

4. How often did you get calls from pharmacists or ID physicians about changing medications to reduce the risk of C. diff?
    a. What did you think of the calls from pharmacists and ID physicians?
    b. How often did you make the recommended medication changes? What kind of medication changes are most common?

5. Have you placed a beta-lactam allergy consult after receiving an alert?
    a. Do you think that the C diff prevention project has had any impact on placing beta-lactam allergy consults for your patients? why/why not.

6. Have you ever encouraged a patient identified as high risk for C diff to eat yogurt?
    a. Do you think that the C diff prevention project has had any impact on patients' ordering yogurt? Why/why not.

7. What questions did you get from patients and visitors about the C diff prevention project [signs, yogurt, hand out], if any?

8. How would you feel about continuing the C diff prevention initiative in the hospital?
    a. Is there anything that would make it easier for your team?

Those are most of my questions. Is there anything else you'd like to tell us about the C difficile prevention program?

Thank you.

© 2025 Tang S et al. *JAMA Network Open.*

ID / PharmD Interview Guide – CDI risk prediction

Introduction / Consent
Hi, my name is [interviewer name] and I'll be leading the interview. Thank you so much for sharing your time with us today. Before we begin, I have some consent information to read through and then I'll give you the chance to ask any questions you might have about the study or the consent process.
[Review Information Sheet]
Do you have any questions for us about the interview or the consent process?
Okay, I am going to turn the recorder on now and ask for your consent so that we have it on record.
TURN RECORDER ON
[Upload to cloud for transcripts to be generated]
For the record, do we have your permission to conduct and audio-record this interview?
[If YES – continue to questions]
[If NO – stop recorder and ask if it is okay to complete the interview without recording if study team only takes notes. If subject agrees, continue to questions and make note that interview was not audio recorded per subject request.]

Interview Questions
Our goal in this interview is to learn more about your experience with an initiative that your unit is participating in to reduce the risk of C difficile infections in patients who are at high risk. A team at Michigan Medicine has developed a prediction model to identify these patients who are at high risk, and for those patients there are several strategies we use to decrease their risk, including [pharmacists' reviewing the appropriateness of antimicrobial agents for these patients / infectious disease specialists' reviewing treatment plans for these patients]. We are interested in learning about your experience as part of this initiative.

1. How long have you been involved in the C difficile prevention project?

2. Can you tell me about your role on the C diff prevention project?
    a. Could you tell me step by step what's involved in this role?
    b. How long does the process take per patient?
    c. How many patients receive this intervention per day?
    d. After the initial intervention, is there any part of the process that involves following up on the patients afterward?

3. What sorts of clinical recommendations do you provide to the clinical teams?

4. Can you describe your interactions with the inpatient teams?
    a. How often do they comply with the recommendations?

5. What barriers if any have you encountered while working on this project?
    a. How were you able to overcome them?

6. Has the role of [pharmacists / infectious disease specialists] changed over the course of the project?
    a. Why were those changes made?
    b. How did those changes work out?

7. What types of efforts were made to educate your colleagues or the inpatient clinical teams when this initiative first rolled out?
    a. How were those received?

8. What has worked well?

9. What hasn't worked so well?

10. Has the time you've invested in this initiative taken away from other important work that you do?

11. Would you recommend that this program continue?
    a. Why / why not?

Those are most of my questions. Is there anything else you'd like to tell us about C diff prevention project?

Thank you.

© 2025 Tang S et al. *JAMA Network Open.*

<u>RN Interview Guide</u> – CDI risk prediction

Introduction / Consent
Hi, my name is [interviewer name] and I'll be leading the interview. Thank you so much for sharing your time with us today. Before we begin, I have some consent information to read through and then I'll give you the chance to ask any questions you might have about the study or the consent process.
[Review Information Sheet]
Do you have any questions for us about the interview or the consent process?
Okay, I am going to turn the recorder on now and ask for your consent so that we have it on record.
TURN RECORDER ON
For the record, do we have your permission to conduct and audio-record this interview?
[If YES – continue to questions]
[If NO – stop recorder and ask if it is okay to complete the interview without recording if study team only takes notes. If subject agrees, continue to questions and make note that interview was not audio recorded per subject request.]

Interview Questions
Our goal in this interview is to learn more about your experience with an initiative that your unit is participating in to reduce the risk of C difficile infections in patients who are at high risk. A team at Michigan Medicine has developed a prediction model to identify patients who are at high risk for C. diff, and for those patients there are several strategies we use to decrease their risk. The piece you may be most familiar with is a quality improvement project sign, printed on brown or white paper, that encourages handwashing with soap and water on room entry. In addition, there is an automated medication review performed by a pharmacist to ensure that patients are on appropriate antibiotics, and patients are encouraged to eat yogurt.

1. To get started, could you tell us a bit about the unit where you work?
    a. How long have you worked there?
    b. How many patients do you generally care for during a shift?
    c. What is the patient acuity?

2. How familiar are you with the C difficile prevention program?
    a. How often do you have patients who have the quality improvement project handwashing sign posted?

3. What is your experience like with patients who are flagged as being at increased risk for C. diff with respect to handwashing?
    a. How easy or difficult is it to wash your hands on room entry?
    b. What were the biggest challenges with handwashing when entering the room?
    c. What do you think would make it easier to wash your hands for patients with a quality improvement project sign posted?
    d. We know there are many different types of staff entering patient rooms (physicians, food service, housekeeping). Do you have a sense of how well others are doing with this hand washing process?

4. Have these handwashing signs changed how you to about your work in any way?
    a. Do you ever go into the room less often than you might have otherwise?
    b. Have you ever experienced nursing assignment changes to prevent nurses who had a C diff patient from also working with one of the quality improvement project (brown-signed) patients?

5. [Questions to charge nurses]
    a. Were there any modifications to how the initiative was done on your unit?
    b. Does this affect your staffing (pairing high-risk patients not with patients are known to have an active infection)?

6. What questions did you get from patients and visitors, if any?

7. Did you find that the C diff prevention project has had any impact on patients' ordering yogurt?

8. How easy or difficult would it be to continue with the C diff prevention initiative on your unit?
    a. Is there anything that would make it easier to continue the initiative?

Those are most of my questions. Is there anything else you'd like to tell us about the C difficile prevention program?

Thank you.

© 2025 Tang S et al. *JAMA Network Open.*

© 2025 Tang S et al. *JAMA Network Open.*

eAppendix 2. Field Observation Recording Templates.

Template for electronic field notes, one note for each room observed, save your document using the following label: Unit & Observation Date (Room 1, 2, 3 if multiple rooms are observed on the same day) Initials (Example: UM UNITX Med Surg 1_2_34 Room X)<br>**DATE:** mm/dd/yyyy<br>**UNIT:**<br>**OBSERVER:** initials<br>**UNIT OBSERVATION DURATION:** time on/time off unit<br>**ROOM OBSERVATION DURATION:** time start/stop<br>**ROOM:** Room #X<br>**GENERAL OVERVIEW:** Include here # rooms on the unit with study enhanced handwashing signs (not sure if we want actual room numbers as that could be considered ‘identifiable’ but might be fine if there is nothing more specific that would link the room to the study.) Note down specific information with time stamps.

Date	Time	Unit	Room Number	Sign on door	No sign on door	Other Notes
		UNIT	ROOM1			
		UNIT	ROOM2			
		UNIT	ROOM3			
		UNIT	ROOM4			
		UNIT	ROOM5			

Top: Recording template for July 2023 field observations. Bottom: Recording template for December 2023 field observations.

© 2025 Tang S et al. *JAMA Network Open.*

eReferences

1. Ravikumar R, Arora NS, Hanson R, et al. A novel 2-step process for the management of inpatient beta-lactam allergy labels. *Annals of Allergy, Asthma & Immunology*. 2024;132(4):525-531. e1.
2. Hickson M, D'Souza AL, Muthu N, et al. Use of probiotic Lactobacillus preparation to prevent diarrhoea associated with antibiotics: randomised double blind placebo controlled trial. *Bmj*. 2007;335(7610):80.
3. CDC National Healthcare Safety Network. Chapter 15: CDC Locations and Descriptions and Instructions for Mapping Patient Care Locations. https://www.cdc.gov/nhsn/pdfs/pscmanual/15locationsdescriptions_current.pdf
4. Quan H, Sundararajan V, Halfon P, et al. Coding Algorithms for Defining Comorbidities in ICD-9-CM and ICD-10 Administrative Data. *Medical Care*. 2005;43(11):1130-1139. doi:10.1097/01.mlr.0000182534.19832.83
5. Valderas JM, Starfield B, Sibbald B, Salisbury C, Roland M. Defining comorbidity: implications for understanding health and health services. *The Annals of Family Medicine*. 2009;7(4):357-363.
6. Furuya-Kanamori L, Stone JC, Clark J, et al. Comorbidities, Exposure to Medications, and the Risk of Community-Acquired Clostridium difficile Infection: A Systematic Review and Meta-analysis. *Infection Control & Hospital Epidemiology*. 2015;36(2):132-141. doi:10.1017/ice.2014.39
7. Affairs DoV. National Drug File - Technical Manual. https://www.va.gov/vdl/documents/Clinical/Pharm-National_Drug_File_(NDF)/psn_4_tm_r0206.pdf
8. Liu S, Wei M, Moore R, Ganesan V, Nelson S. RxNorm: prescription for electronic drug information exchange. *IT Professional*. 2005;7(5):17-23. doi:10.1109/MITP.2005.122
9. Michigan Medicine Antimicrobial Stewardship. Guideline for the Treatment of *Clostridioides difficile* in Adult Patients. https://antimicrobialstewardship.med.umich.edu/guidelines/adult/cdiff-treatment
10. Michigan Medicine Laboratories. *C. difficile* PCR/toxin algorithm | MLabs. https://mlabs.umich.edu/tests/c-difficile-pcrtoxin-algorithm
11. Michigan Medicine Laboratories. *Gastrointestinal Pathogen Panel* | MLabs. https://mlabs.umich.edu/tests/gastrointestinal-pathogen-panel
12. CDC National Healthcare Safety Network. Chapter 12: MDRO & CDI Module Protocol – January 2024. https://www.cdc.gov/nhsn/pdfs/pscmanual/12pscmdro_cdadcurrent.pdf
13. CDC National Healthcare Safety Network. Chapter 14: Antimicrobial Use and Resistance (AUR) Module – January 2024. https://www.cdc.gov/nhsn/pdfs/pscmanual/11pscaurcurrent.pdf
14. CDC National Healthcare Safety Network. Determining Patient Days for Summary Data Collection: Observation vs. Inpatients. https://www.cdc.gov/nhsn/pdfs/commup/patientday_sumdata_guide.pdf
15. Michigan Medicine Antimicrobial Stewardship. Treatment Pathways for Adult Patients with Pneumonia. https://antimicrobialstewardship.med.umich.edu/guidelines/adult/pneumonia-treatment

© 2025 Tang S et al. *JAMA Network Open.*
