# Supplementary material for: Guiding Clostridioides difficile Infection Prevention Efforts in a Hospital Setting With AI
Source: JAMA Netw Open. 2025 Jun 12;8(6):e2515213. doi: 10.1001/jamanetworkopen.2025.15213 (PMC12163649; doi:10.1001/jamanetworkopen.2025.15213)
Supplement: Supplement 2. — Data Sharing Statement [file jamanetwopen-e2515213-s002.pdf]

Data Sharing Statement

Tang. Guiding Clostridioides difficile Infection Prevention Efforts in a Hospital Setting With AI. *JAMA Netw Open*. Published June 12, 2025. doi:10.1001/jamanetworkopen.2025.15213

Data

Data available: No
